# High glucose promotes the progression of colorectal cancer by activating the BMP4 signaling and inhibited by glucagon-like peptide-1 receptor agonist

**DOI:** 10.1186/s12885-023-11077-w

**Published:** 2023-06-27

**Authors:** Bingwei Ma, Xingchun Wang, Hui Ren, Yingying Li, Haijiao Zhang, Muqing Yang, Jiyu Li

**Affiliations:** 1 Colorectal Cancer Central, Shanghai Tenth People’s Hospital, Tongji University School of Medicine, 301 Middle Yanchang Road, Shanghai, 200072 China; 2 Department of Endocrinology and Metabolism, Shanghai Tenth People’s Hospital, Tongji University, 301 Middle Yanchang Road, Shanghai, 200072 China; 3 Thyroid Research Center of Shanghai, Shanghai Tenth People’s Hospital, 301 Middle Yanchang Road, Shanghai, 200072 China; 4 School of Pharmacy, East China University of Science and Technology, 130 Meilong Road, Shanghai, 200237 China; 5 Department of Gastrointestinal Surgery, Huadong Hospital affiliated with Fudan University, 221 West Yanan Road, Shanghai, 200040 China; 6 Department of General Surgery, Tenth People’s Hospital of Tongji University, 301 Middle Yanchang Road, Shanghai, 200072 China; 7 Geriatric Cancer Center, Huadong Hospital Affiliated to Fudan University, 221 West Yanan Road, Shanghai, 200040 China

**Keywords:** T2DM, Colorectal cancer, BMP4, EMT, GLP-1RA

## Abstract

**Background:**

The detailed molecular mechanism between type 2 diabetes mellitus (T2DM) and colorectal cancer (CRC) is still uncertain. Bone morphogenetic protein 4 (BMP4) dysregulation is implicated in T2DM and CRC, respectively. This study aims to investigate whether BMP4 can mediate the interaction of CRC with T2DM.

**Methods:**

We firstly explored the expression of BMP4 in The Cancer Genome Altas (TCGA) databases and CRC patients with or without DM from the Shanghai Tenth People’s Hospital. The diabetic model of CRC cell lines in vitro and the mice model in vivo were developed to explore the BMP4 expression during CRC with or without diabetes. Further inhibition of BMP4 to observe its effects on CRC. Also, glucagon-like peptide-1 receptor agonist (GLP-1RA) was used to verify the underlying mechanism of hypoglycemic drugs on CRC via BMP4.

**Results:**

BMP4 expression was upregulated in CRC patients, and significantly higher in CRC patients with diabetes (P < 0.05). High glucose-induced insulin resistance (IR)-CRC cells and diabetic mice with metastasis model of CRC had increased BMP4 expression, activated BMP4-Smad1/5/8 pathway, and improved proliferative and metastatic ability mediated by epithelial-mesenchymal transition (EMT). And, treated CRC cells with exogenously BMP inhibitor-Noggin or transfected with lentivirus (sh-BMP4) could block the upregulated metastatic ability of CRC cells induced by IR. Meanwhile, GLP-1R was downregulated by high glucose-induced IR while unregulated by BMP4 inhibitor noggin, and treated GLP-1RA could suppress the proliferation of CRC cells induced by IR through downregulated BMP4.

**Conclusions:**

BMP4 increased by high glucose promoted the EMT of CRC. The mechanism of the BMP4/Smad pathway was related to the susceptible metastasis of high glucose-induced IR-CRC. The commonly used hypoglycemic drug, GLP-1RA, inhibited the growth and promoted the apoptosis of CRC through the downregulation of BMP4. The result of our study suggested that BMP4 might serve as a therapeutic target in CRC patients with diabetes.

**Supplementary Information:**

The online version contains supplementary material available at 10.1186/s12885-023-11077-w.

## Background

Colorectal cancer (CRC), one of the most frequent and deadly malignancies, is the third most commonly diagnosed cancer among all types of cancers and the second cause of cancer death [1]. Many factors associated with the onset of CRC have been found and investigated, including genetic reason, lifestyle, diet habits, chronic diseases, and so on [2]. Diabetes mellitus (DM), one of the most common chronic diseases, has been reported to be associated with many kinds of cancer, like colorectum, lung, pancreas, esophagus, liver, thyroid, breast, and so on [3, 4]. There may exist direct or indirect associations between DM and cancer, and no one can deny that much has been done in molecular research [5]. However, the detailed association and mechanism are still unclear, and relative studies about type 2 diabetes mellitus(T2DM) and CRC are still worthy of investigation.

Bone Morphogenetic Proteins (BMPs), one of the largest subgroups of the transforming growth factor-β (TGF-β) superfamily, were initially found can induce mesenchymal stem cells to differentiate into bone and promote the formation of bone and cartilage formation [6]. Besides, emerging evidence has proved that BMPs are multifunctional and involved in many physiological regulatory processes, including cell differentiation, apoptosis, immunology, metabolism, and so on [7–10]. BMPs play a function mainly through the canonical and noncanonical pathways. The canonical signaling pathway, also known as SMAD dependent pathway, in which the secreted BMPs bind with heteromeric complexes of type I receptor(BMPRI) and type II receptor(BMPRII) to activate the canonical signal transduction pathway [11]. Both receptor types are serine/threonine kinases, and have a short extracellular domain, a single transmembrane domain, and an intracellular domain. The combination of BMPs with BMPR furtherly activates the phosphorylation of the Smad1/5/8 complex, which is assembled with Smad4 and is transferred into the nucleus to activate or suppress the expression of relative genes [12]. Besides the canonical signaling pathway, BMPs also can function through non-canonical BMP pathways like extracellular signal-regulated protein kinase(ERK)/MAP kinase(MAPK) and phosphoinositide 3-kinase (PI3K)/protein kinase B (AKT) pathway [13, 14].

Studies have reported that BMP4 is implicated in the progression of many types of cancer, like breast, colon, bladder, and gastric cancer [15–17]. The upregulated expression of BMP4 has been found in CRC and promotes the progression of carcinoma [18]. Epithelial-mesenchymal transition (EMT) as one of the most important mechanisms in cancer metastasis also has been reported by many studies that could be induced by BMP4 in many kinds of cancer [17, 19]. In addition, BMP4 plays a vital role in metabolic diseases and is markedly increased in patients with impaired glucose tolerance or T2DM [20–22]. Serum BMP4 levels are negatively associated with insulin sensitivity [22]. And BMP4 expression increased in diabetic animals reduces glucose-stimulated insulin secretion [23] and inhibits beta cell growth and function [24]. However, no study investigated whether the glucose metabolic disorder in CRC interacted with BMP4 to affect the progression of CRC.

Considering that BMP4 may crosstalk glucose metabolism and CRC, we designed this study to investigate whether the increased BMP4 induced by diabetes facilitates the progression of CRC, which may contribute to finding a potential therapeutic target for the treatment of CRC. In addition, we will further explore whether glucagon-like peptide-1 receptor agonist(GLP-1RA), a widely used drug for T2DM, can affect the progression of CRC by meditating the expression of BMP4 [25].

## Methods

### Patients and demographic data

In this study, 37 CRC patients who underwent laparoscopic colorectal surgery were enrolled between Jan 2021 and May 2021 from the Shanghai Tenth People’s Hospital. According to with or without the comorbidities of T2DM, patients were divided into two groups the CA + DM group(with T2DM) and the CA group(without T2DM). The diagnosis of T2DM based on the diagnosis of a physician and/or prescription of glucose-lowering drugs and/or abnormal laboratory values as defined by the American Diabetes Association guidelines: HbA1c ≥ 6.5%, or fasting plasma glucose ≥ 7.0 mmol/L, or 2-h plasma glucose ≥ 11.1 mmol/L during a 75 g oral glucose tolerance test (OGTT), or random plasma glucose > 11.1 mmol/L with typical symptoms of hyperglycemia or hyperglycaemic crisis [26]. Blood samples of these patients were collected during the first 24 h after hospital admission. Blood was collected in an EDTA anticoagulant tube. After centrifugation at 3000 rpm for 10 min, the upper plasma specimens were collected and stored at -80℃. In addition, the demographic data of enrolled patients were collected from the electronic record, including age, sex, height, weight, BMI (body mass index), tumor size, tumor node metastasis (TNM) stage, and tumor differentiation. Additionally, plasma specimens were obtained as described above from 37 patients undergoing CRC resection. The Human BMP4 ELISA Kit (ab231930, Abcam, USA) was used to determine plasma BMP4 levels as per manufacturer instruction, and the result was reported in pg/ml. All specimens were tested in duplicate. The protocol was approved by the Ethics Committee of the Shanghai Tenth People’s Hospital.

### Cell culture and insulin resistance model of CRC cell

The human colorectal cell lines HCT116, SW480, SW620, and SW1116 were obtained from the Chinese Academy of Sciences cell bank. MC38 cell line was purchased from ATCC (CL0203). These cells were cultured in RPMI-1640 medium supplemented with 10% fetal bovine serum (FBS) and 1% penicillin and streptomycin at 37 °C in 5% CO2. The insulin resistance (IR) model of CRC cells was cultured with RPMI-1640 medium containing 50mM concentration glucose for 24 h. A Glucose consumption assay was used to verify the establishment of IR. CRC cell lines (1*10^5^ cells/mL) were seeded in 96-well plates and treated with high glucose. A glucose assay kit was supplied from Nanjing Jiancheng Bioengineering Institute (Nanjing, China), which was used to measure glucose concentrations in the culture medium. The culture medium was collected, and glucose reagent was added and then placed at 37 °C for 15 min. The OD values were measured at 505 nm. The results were calculated as follows: Glucose consumption = glucose concentration in the blank group (no cells, only medium) – glucose concentration in each group (model and control groups)[27]. The relative levels of glucose consumption in cells were normalized to that of the control group.

### Externally intervention and lentiviral transfection

Noggin (200nM)(ab281817, Abcam, USA), an inhibitor of BMP4, and active recombinant human BMP4 (100nM)(ab238298, Abcam, USA) were added to cell culture for 48 h for inhibition or overexpression of BMP4 respectively. In addition, we used the lentiviral vector system (Genomeditech Biotechnology Co. Ltd, Shanghai, China) transfected SW1116 cells to establish the BMP4 downregulation model(shBMP4-SW1116). Finally, western blotting was used to validate the efficiency of BMP4 downregulation.

### Cell viability assay

Cell viabilities were determined by cell counting kit-8 (CCK-8, Nanjing Jiancheng, China) assay, and the CRC cells (1*10^4^ cells/well) were seeded on 96-well plates [27]. To determine high glucose-induced IR to promote the proliferation of CRC cells, cells were serum starved for 12 h and then treated with high glucose(50mM) and recombinant Noggin (200nmol/L) for 48 h. To investigate the effect of GLP-1RA, liraglutide, on the growth of CRC cells, the cells were treated with serum starvation for 12 h and then incubated with liraglutide (NovoNordisk, Bagsvaerd, Denmark) at 0, 10, 20, 50,100nM and the combination of liraglutide(50nM) and recombinant BMP4(Abcam, 100nM) for 48 h. After incubation, each cell was added with 10 µL CCK-8 solution and furtherly incubated for 2 h at 37 °C and 5% CO2, and the absorbance was measured at 450 nm by an ELISA reader (SpectraMax iD5, USA).

### Transwell assay

Each cell culture insert (Corning, Tewksbury, MA) containing culture media without serum was seeded with 1.5 × 10^5^cells, and the stimuli were added to the lower chambers. After 24 h, take out the insert, and the wet cotton swab was used to remove the non-migrated cells on the top side of the filter. Next, 4% paraformaldehyde (PFA) (Solarbio, China) was used to fix the migrated cells on the bottom side of the filter for 30 min. Then stained the migrated cells with 0.1% crystal violet solution (Solarbio, China) for 20 min and washed three times with PBS. After thoroughly drying, visualized the migrated cells under a microscope.

### RNA isolation and quantitative real-time PCR (qRT-PCR)

Total RNA was derived from cells using RNAsimple Kit (Tiangen) following the manufacturer’s instructions, and complementary DNA (cDNA) was synthesized from 1000 ng of total RNA using the Reverse Transcription Kit (Takara Bio) for qRT-PCR. qRT-PCR was performed using a 7900 H T real-time PCR system (ABI, CA, USA) to determine the expression of target genes according to the instructions of SYBR Green Master Mix (KAPA Japan) for quantitative PCR. In addition, Glyceraldehyde 3-phosphate dehydrogenase (GAPDH) was used as an endogenous control for gene expression and was analyzed using the △△-Ct method. The primer sequences for the genes analyzed are listed in Table 1.

**Table 1:** qRT-PCR primer sequences

Gene	Forward sequence	Reverse sequence
BMP4 (human)	5’- GGAGGAGGAGGAAGAGCAGA-3’	5’- CACTGGTCCCTGGGATGTTC-3’
BMP4 (mouse)	5’- ATTCCTGGTAACCGAATGCTG-3’	5’- CCGGTCTCAGGTATCAAACTAGC-3’
GAPDH (human)	5’- CATGAGAAGTATGACAACAGCCT-3’	5’- AGTCCTTCCACGATACCAAAGT-3’
GAPDH (mouse)	5’- CCTGCACCACCAACTGCTTA-3’	5’- TCAGATCCACGACGGACACA-3’

qRT-PCR, real-time reverse-transcriptase polymerase chain reaction; BMP4, bone morphogenetic protein 4

### Western blotting (WB)

Proteins of HCT116, SW480, SW620, SW1116, MC38, and SW1116-shBMP4 cells and tumor tissues were extracted and lysed in 10% SDS. Cell lysates were the first subject to SDS-PAGE, transferred to polyvinylidene fluoride membranes (Millipore, USA), and then blocked with 5% non-fat milk. Primary antibodies, including BMP4 (1:1000, ab124715, Abcam, USA), BMP4(1:1000, ab33973, Abcam, USA), E-cadherin (1:5000, 20874-1, Proteintech, China), Vimentin (1:5000, 60330-1, Proteintech, China), N-cadherin (1:5000, 66219-1, Proteintech, China), cleaved-caspase3(1:1000, TA7022, Abmart, China), BCL-2(1:1000, T40056, Abmart, China), Snail(1:1000, 13099-1, Proteintech, China), GAPDH(1:50000, 60004-1, Proteintech, China), β-Tublin(1:5000, M20023, Abmart, China) were added to the membranes at 4 °C overnight. The next day, the secondary antibodies, including goat anti-rabbit IgG-HRP (1:1000, ZB-2301, Zhongshan Chemical, China), goat anti-mouse IgG-HRP (1:1000, ZB-2305, Zhongshan Chemical, China) were added for 1 h at room temperature. The protein levels were assessed using the enhanced chemiluminescence method.

### Immunohistochemistry (IHC)

Detail procedure of IHC followed the previous study [28]. First, paraffin-embedded tumor samples (5-µm) were deparaffinized, hydrated, and treated with endogenous peroxidases. Then, the slides were subject to antigen retrieval. Next, the slides were subject to 10% normal goat serum to inactivate endogenous peroxidase and treated overnight with rabbit monoclonal against BMP4 antibody (ab124715, Abcam, USA) at 4 °C. Then washed with PBS three times and subsequently incubated with a secondary antibody at room temperature for 1 h. After incubation, furtherly performed hematoxylin counterstain.

### Flow cytometric analysis

The Annexin V-FITC/PI Apoptosis Detection Kit (Multisciences, Hangzhou, China) was used to assay the apoptosis of the SW1116 cell line treated with different concentrations of GLP-1RA. According to the manufacturer’s protocol, cells and cultural supernatants were collected and centrifuged at 1000 rpm for 5 min. After centrifugation, removed the supernatants carefully and washed the cell twice with cold PBS. Re-suspended the cell pellets in 500µL of binding buffer. Then, PI solution (5µL) and FITC labeled Annexin V (5µL) were incubated with cells in the dark for 5 min. A flow cytometer (Becton Dickinson, USA) was used to analyze the sample. The proportion of cells in each quadrant was furtherly analyzed with FlowJo software.

### High-fat diet/streptozotocin (HFD/STZ)-induced mouse models of T2DM

This study followed the Guide for the Care and Use of Laboratory Animals of the National Institutes of Health. The protocol was approved by the Institutional Animal Care and Use Committee of the Shanghai Tenth People’s Hospital. Male C57BL/6J mice (4–6 weeks old) were purchased from the Shanghai Laboratory Animal Center (Shanghai, China). They were housed under a 12/12-h light/dark cycle at 19 to 23 °C. The mice were randomly divided into a control group(n = 5) and a diabetic group(n = 5). Mice of diabetes received 5 weeks HFD (a diet with 60% kcal from fat) (Medicience Ltd., Jiangsu, China) firstly, then a consecutive five days intraperitoneal injection with a freshly prepared solution of STZ (Sigma-Aldrich, St Louis, MO, USA) at 40 mg/kg body weight. Three days after the injection, we measured the random blood glucose levels of the mice, and mice with a blood glucose level of 16.7mmol/L or higher were considered to have T2DM [29]. The mice in the control group were fed a regular chow diet and intraperitoneal injection with citrate-phosphate buffer.

### In vivo xenograft studies

The process was referenced in the previous study [30]. Log-phase cells were harvested with 0.05% trypsin–0.02% EDTA in MC38 cells Hanks Balanced Salt Solution (HBSS), washed three times with PBS, and suspended in PBS at a final concentration of 5*10^7^ cells/ml. The C57BL/6 mice were anesthetized with an intraperitoneal injection of pentobarbital (Nembutal, Dainippon Sumitomo Pharma Co., Ltd., Osaka) regulated to 75 mg/kg. Then the mice were incised about 10 mm on the left subcostal, the spleen was confirmed under the peritoneum, the peritoneum was opened for about 8 mm, and the spleen was exposed over the peritoneum. Next, a needle injected the cell suspension of 5*10^6^/100ul of human colon cancer cells into the spleen. After 5 min, the spleen was resected, the peritoneum was sutured with one stitch, and the wound was closed with a clip [31]. The mice were killed two weeks after inoculation with tumor cells, and the liver sample was resected for evaluation. The tumor sections were paraffin-embedded and preserved for immunohistochemical analysis. All mice experiments were performed with the approval of the Animal Ethics Committee of Tongji University (approval number: SHDSYY-2021-2965a).

### Statistical analysis

SPSS22.0 software was used to perform statistical analysis. For continuous variables, data were presented as mean with standard deviation (normal distribution) and median with interquartile range (IQR)(non-normal distribution). In addition, an independent-sample t-test and relative values using a two-tailed Fisher exact test were used for continuous data. For categorical variables, data were summarized as counts with percentages, and the Pearson correlation analysis was performed. All the tests were two-sided, and the value of p < 0.05 was considered statistically significant.

## Results

### Expression of BMP4 was upregulated in CRC and associated with poor outcome

**Fig. 1:**
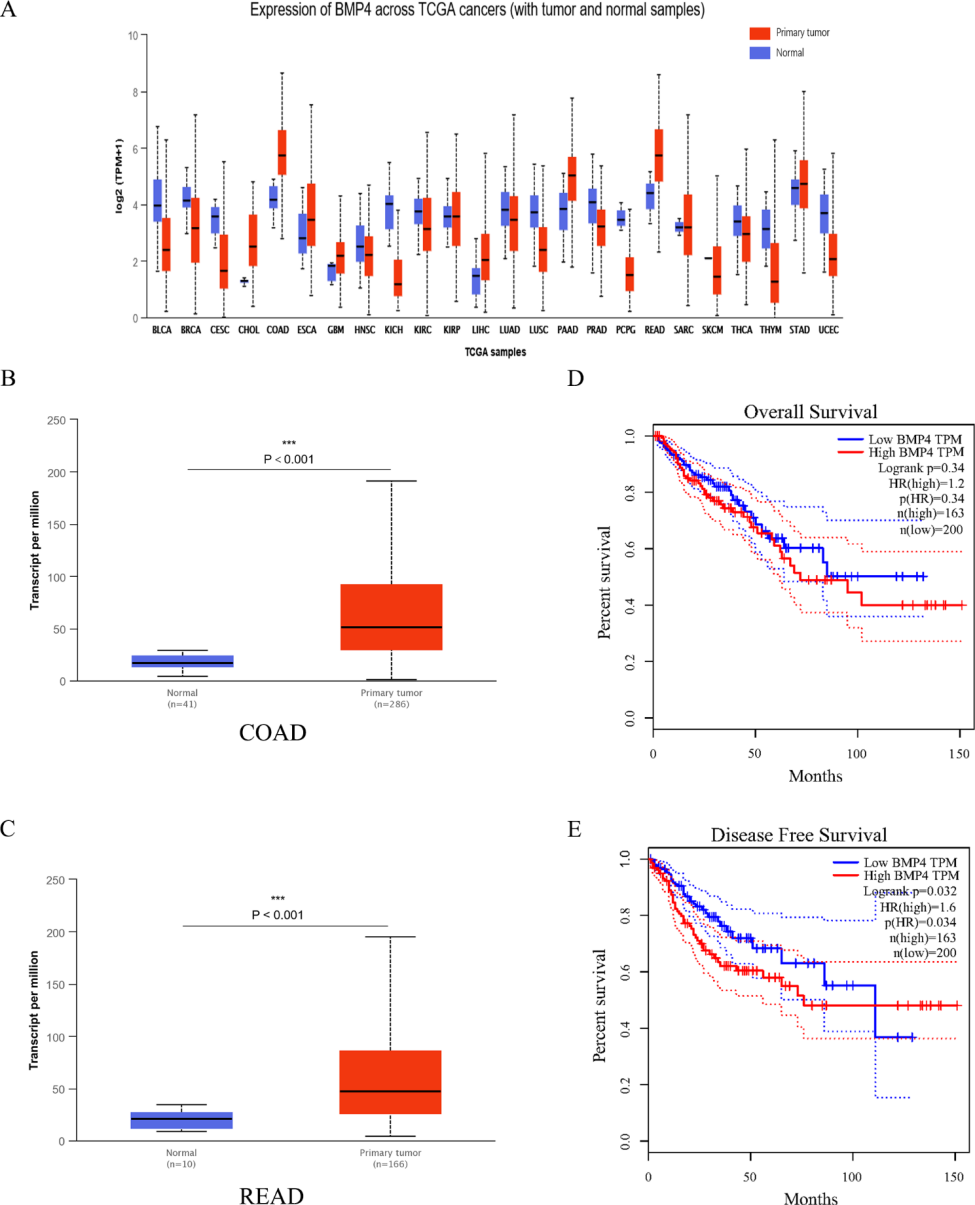
The expression of BMP4 was increased in CRC according to the data from the TCGA database (**A**) BMP4 expression was investigated in different types of cancer in the TCGA database (**B**, **C**) The expression of BMP4 was significantly increased in COAD(**B**) and READ(**C**) tumor samples(all P < 0.01) in the TCGA database (**D**, **E**) The expression of BMP4 was negatively associated with overall survival probability and disease free survival probability(all p < 0.05) BLCA, Bladder Urothelial Carcinoma; BRCA, Breast invasive carcinoma; CESC, Cervical squamous cell carcinoma and endocervical adenocarcinoma; CHOL, Cholangio carcinoma; COAD, Colon adenocarcinoma; ESCA, Esophageal carcinoma; GBM, Glioblastoma multiforme; HNSC, Head and Neck squamous cell carcinoma; KICH, Kidney Chromophobe; KIRC, Kidney renal clear cell carcinoma; KIRP, Kidney renal papillary cell carcinoma; LIHC, Liver hepatocellular carcinoma; LUAD, Lung adenocarcinoma; LUSC, Lung squamous cell carcinoma; PAAD, Pancreatic adenocarcinoma; PRAD, Prostate adenocarcinoma; PCPG, Pheochromocytoma and Paraganglioma; READ, Rectum adenocarcinoma; SARC, Sarcoma; SKCM, Skin Cutaneous Melanoma; THCA, Thyroid carcinoma; THYM, Thymoma; STAD, Stomach adenocarcinoma; UCEC, Uterine Corpus Endometrial Carcinoma

We conducted an intensive analysis of gene alterations in CRC from the TCGA database using an easy-to-use, interactive web portal, ALCAN, and the correlation between BMP4 gene expression and colorectal cancer clinical features [32]. Through the online database, we identified that the expression of BMP4 was significantly high in colorectal tumor (Fig. 1A). And, BMP4 was significantly upregulated in tumor tissues compared with adjacent normal tissues of CRC (P < 0.001) (Fig. 1B, C). Meanwhile, high-level BMP4 was associated with poor outcomes for CRC patients. Although, the percent of overall survival(OS) did not have a significant difference, the percent of disease free survival (DFS) of the higher BMP4 group was significantly lower than the lower BMP4 group(P = 0.032) (Fig. 1D, E).

### CRC patients with diabetes had higher expression of BMP4

**Fig. 2:**
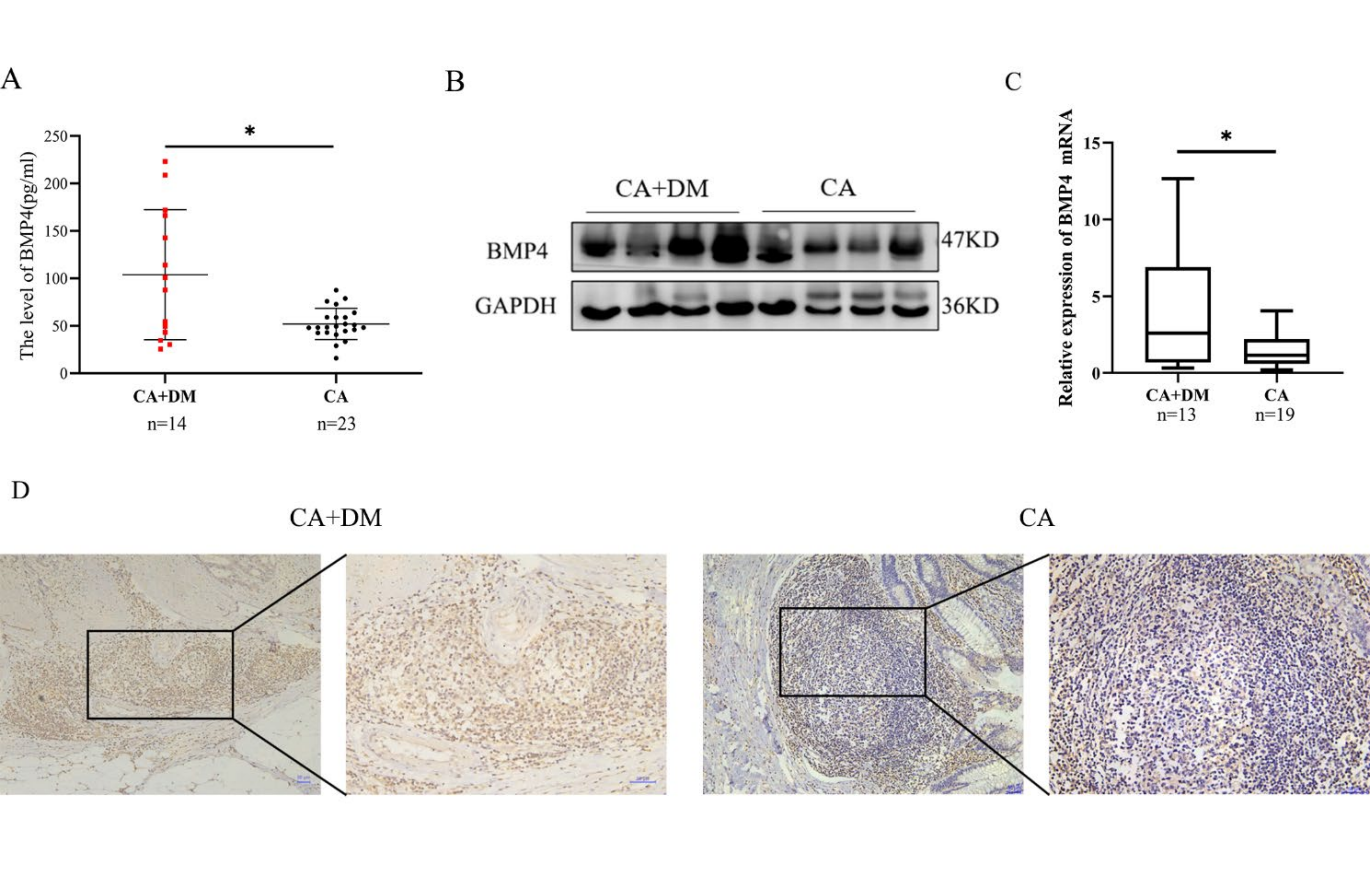
BMP4 expression was significantly higher in CRC patients with DM than in CRC patients without DM. (**A**) The plasma level of BMP4 in CRC patients with DM (CA + DM group) was significantly higher than patients without DM (CA group), *compared to the CA group, P < 0.05 (**B**, **C**) The upregulation of the protein and RNA expression of BMP4 was confirmed by western blotting and qRT-PCR in CRC specimens from patients with or without DM, *compared to the CA group, P < 0.05 (**D**) Immunohistochemical localization of BMP4 in tumor samples of CRC patients with or without DM (scale bar = 50 μm)

Fourteen patients with T2DM were divided into the CA + DM group, and 23 non-diabetic patients with age, height, weight, and sex ratio matched (all P value>0.05) belong to the CA group. Detailed information of these patients was shown in Table 2. The CA + DM group had significantly higher BMP4 level when compared with the group of CA (103.83 ± 68.54 vs. 51.97 ± 16.39pg/ml, P = 0.015) (Fig. 2A).

**Table 2:** The demographic characteristic of CRC patients

	CA group (n = 23)	DM + CA group (n = 14)	P value
Age, years, mean ± SD	67.26 ± 13.68	66.64 ± 8.71	0.881
Sex, n(%) Male Female	11(47.83%) 12(52.17%)	5(35.71%) 9(64.29%)	0.515
Height, cm, mean ± SD	164.35 ± 8.46	164.57 ± 9.06	0.940
Weight, kg, mean ± SD	62.07 ± 14.57	65.76 ± 15.76	0.472
BMI, kg/m^2^, mean ± SD	22.81 ± 4.12	23.97 ± 3.56	0.387
Anemia, n(%) Yes No	15(65.22%) 8(34.78%)	9(64.29%) 5(35.71%)	1.000
Hypoproteinemia, n(%) Yes No	21(91.30%) 2(8.70%)	12(85.71%) 2(14.29%)	0.625
TNM stage, n(%) I/II III/IV	15(65.22%) 8(34.78%)	8(57.14%) 6(42.86%)	0.732
Tumor size ≥ 5 cm, n(%) Yes No	15(65.22%) 8(34.78%)	9(64.29%) 5(35.71%)	1.000
Tumor differentiation, n(%) Low Moderate & High	10(43.48%) 13(56.52%)	7(50.00%) 7(50.00%)	0.481

BMI, body mass index; TNM, tumor node metastasis

The protein levels of BMP4 in tumor tissues (n = 8) from frozen tissue samples were analyzed by western blotting. The results showed that the protein level of BMP4 was higher in patients with diabetes than in patients without diabetes (Fig. 2B). To further investigate the expression difference of BMP4, we also collected 32 CRC tissue specimens for qRT-PCR, and the result showed that BMP4 mRNA was upregulated in CRC patients with diabetes **(**Fig. 2C). Typical IHC images of BMP4 in tumor samples of colorectal cancer patients were shown in Fig. 2D. The results of IHC showed that the expression of BMP4 in tumor tissue was significantly higher in patients with diabetes.

### Glucose consumption in IR of CRC cell lines

**Fig. 3:**
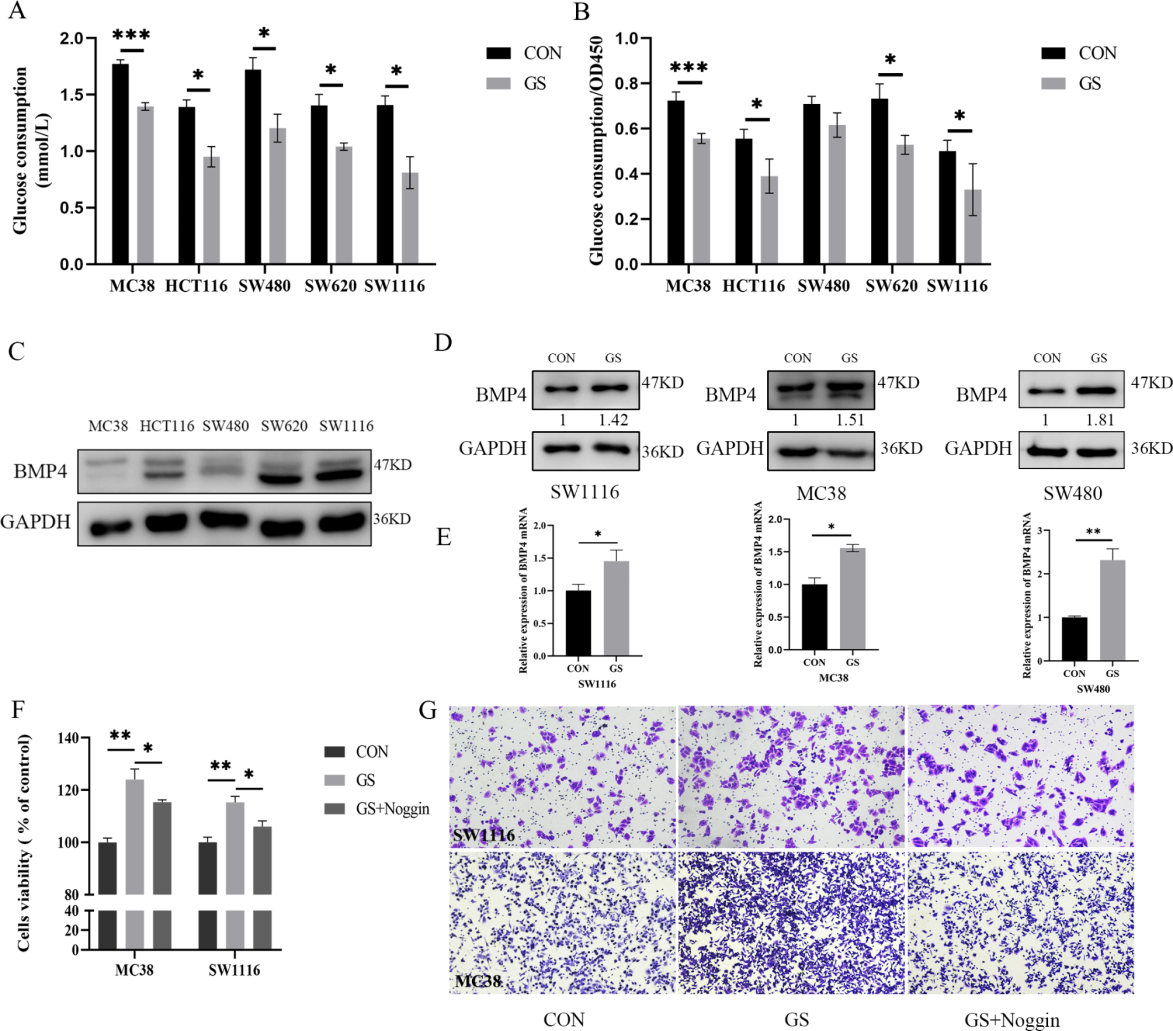
High glucose promoted the proliferation and metastasis of CRC cells (**A**) Glucose consumption was measured in different types of CRC cell lines by glucose assay kit, *P < 0.05, ***P < 0.001 (**B**) The result of glucose consumption was rectified by cell viability measured by CCK-8 kit (OD 450), *P < 0.05, ***P < 0.001 (**C**) Protein of BMP4 expression examined by western blotting in different types of CRC cell lines (**D**, **E**) Treated with high glucose(50mM) 48 h could upregulate the expression of BMP4 measured by western blotting(**D**) and qRT-PCR(**E**), *P < 0.05 (**F**) Cell viability of SW1116 and MC38 cultivated by high glucose(50mM) and Noggin(200nM) for 48 h assessed by CCK-8kit, *P < 0.05, ***P < 0.001 (**G**) Transwell assays were performed to examine the potential migration of SW1116 and MC38 cells treated with or without high glucose(50mM) and Noggin(200nM).

To establish a high glucose-induced IR cell model, we cultured CRC cell lines (MC38, HCT116, SW480, SW6200, SW1116) with the 1640 medium containing 50 mM glucose for 24 h in accordance with the previous protocol [33]. As results shown in Fig. 3A, the glucose consumption of five cell lines treated with high glucose(GS group) was significantly decreased compared with the control group(CON group)(all P<0.05). In order to diminish the effect of cell viability on glucose consumption, we used the value of OD450 to rectify the glucose consumption, and the results were showed in Fig. 3B. Compared with the CON group, the GS group had significantly lower glucose consumption/OD450 indicating the IR-CRC cell model was established.

### BMP4 was upregulated in IR-CRC cells and promoted the migration of CRC cells

We first evaluated the baseline expression level of BMP4 in one mouse-derived cell line (MC38) and four human-derived cell lines (HCT116, SW480, SW620, SW1116) (Fig. 3C). For further investigation, we chose two cell lines in our study, SW1116 and MC38, as represented. The western blotting and qRT-PCR results showed that high glucose-induced IR could elevate the expression of BMP4, as shown in Fig. 3D, E.

To elucidate the effect of high glucose-induced IR on CRC cell proliferation in vitro, cell viabilities were examined by CCK-8 assay. The results showed that high glucose(50mM) significantly promotes the proliferation of MC38 and SW1116. And, this proliferative effect can be significantly diminished by the addition of Noggin (GS + Noggin group), the extracellular inhibitor of BMP4, as shown in Fig. 3F. Furthermore, with the high glucose, the migration of MC38 and SW1116 was significantly improved by the results of Transwell assay (Fig. 3G). And, compared with the GS group, the migration was reduced in the GS + Noggin group. The results above indicated that high glucose could promote the proliferation and metastasis of CRC cell line through upregulated the expression of BMP4.

### High-glucose induced IR regulated the EMT of CRC cells through BMP4/Smad pathway

**Fig. 4:**
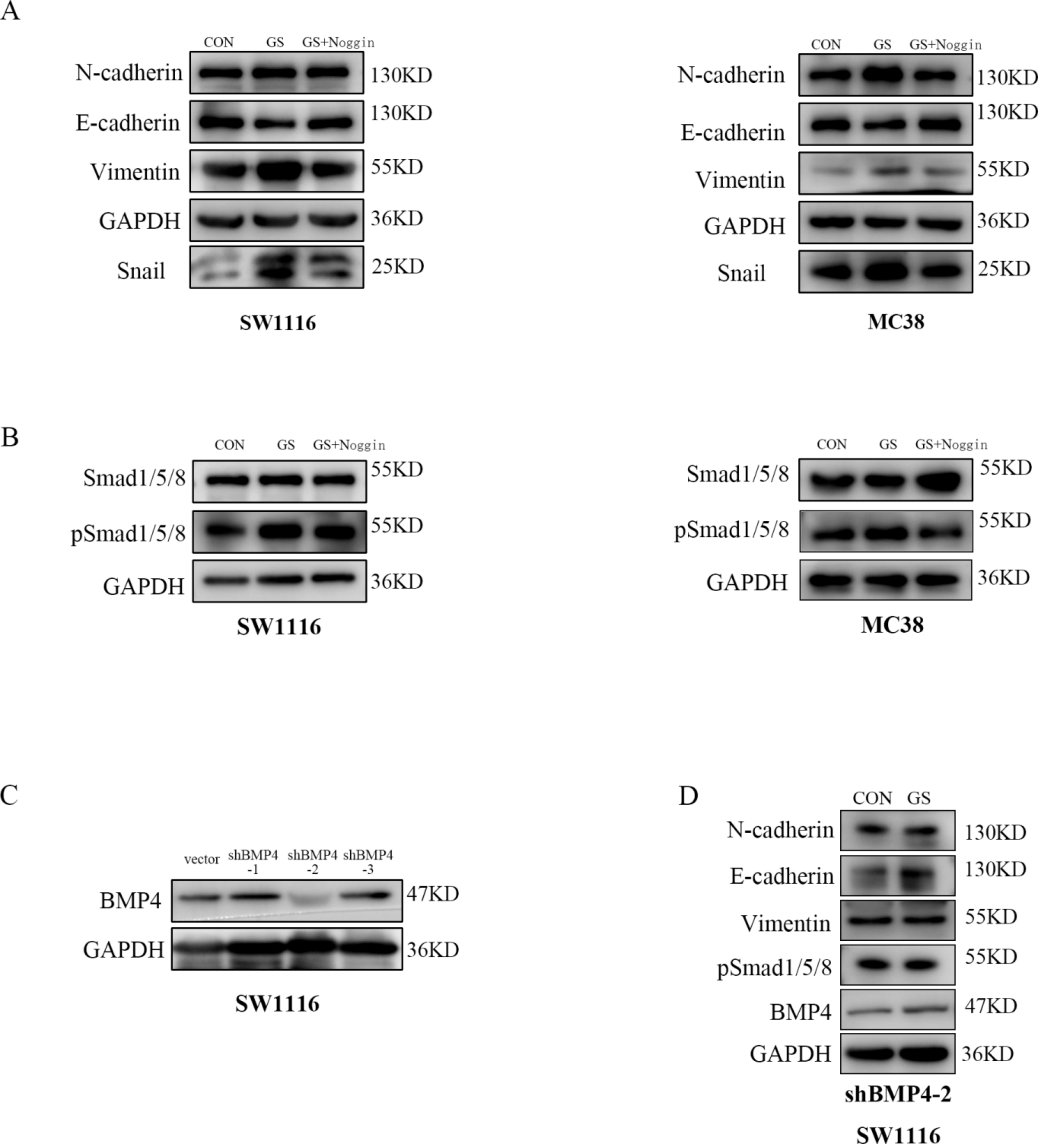
High glucose promoted the EMT of SW1116 and MC38 cells through the BMP4-Smad pathway (**A**) Western blotting analysis of the expression of EMT markers in SW1116 and MC38 cells cultivated with or without high glucose(50mM) and Noggin(200nM). (**B**) High glucose activated the SMAD1/5/8 and this effect could be blocked by Noggin proved by western blot analysis (**C**) Western blotting analysis of the efficiency of sh-BMP4 and vector transfection in SW1116 cells (**D**) Western blotting analysis of the expression of BMP4, EMT markers and pSMAD1/5/8 in SW1116 transfected with sh-BMP4-2 treated with or without high glucose(50mM) for 24 h

We furtherly investigated the expression of markers associated with EMT, a crucial biological step driving tumor cell invasion and metastatic dissemination from primary tumors [34]. Compared with the CON group, the GS group expressed higher protein levels of N-cadherin, Vimentin and Snail while lower E-cadherin (Fig. 4A). As the canonical signaling pathway of BMP4, we investigated whether high glucose induced IR activated pSmad1/5/8 to mediate the EMT [35]. According to the western blotting result, the expression of pSmad1/5/8 was upregulated upon high glucose treated in SW1116 and MC38(Fig. 4B). However, Noggin, the inhibitor of BMP4, could deregulation the expression of pSmad1/5/8 and resistance EMT induced by high glucose (Fig. 4A, B). Specific shRNAs (vector, sh-BMP4-1, 2, 3) were transfected into SW1116 cells, and the transfection efficiency was confirmed by the western blotting results of BMP4 (Fig. 4C). As shown in Fig. 4C, shBMP4-2 has the highest transfection efficiency, and the SW1116 cell line transfected with it was further cultured with or without high glucose (GS vs. CON group). In SW1116 cells transfected with shBMP4-2, the intervention of high glucose did not significantly change the expression of BMP4, EMT markers and pSmad1/5/8 compared with the CON group (Fig. 4D). These results indicated that high-glucose induced IR of CRC cells upregulated the BMP4 and mediated EMT through the canonical Smad pathway.

### GLP-1RA diminished the proliferative of CRC by decreasing the expression of BMP4

**Fig. 5:**
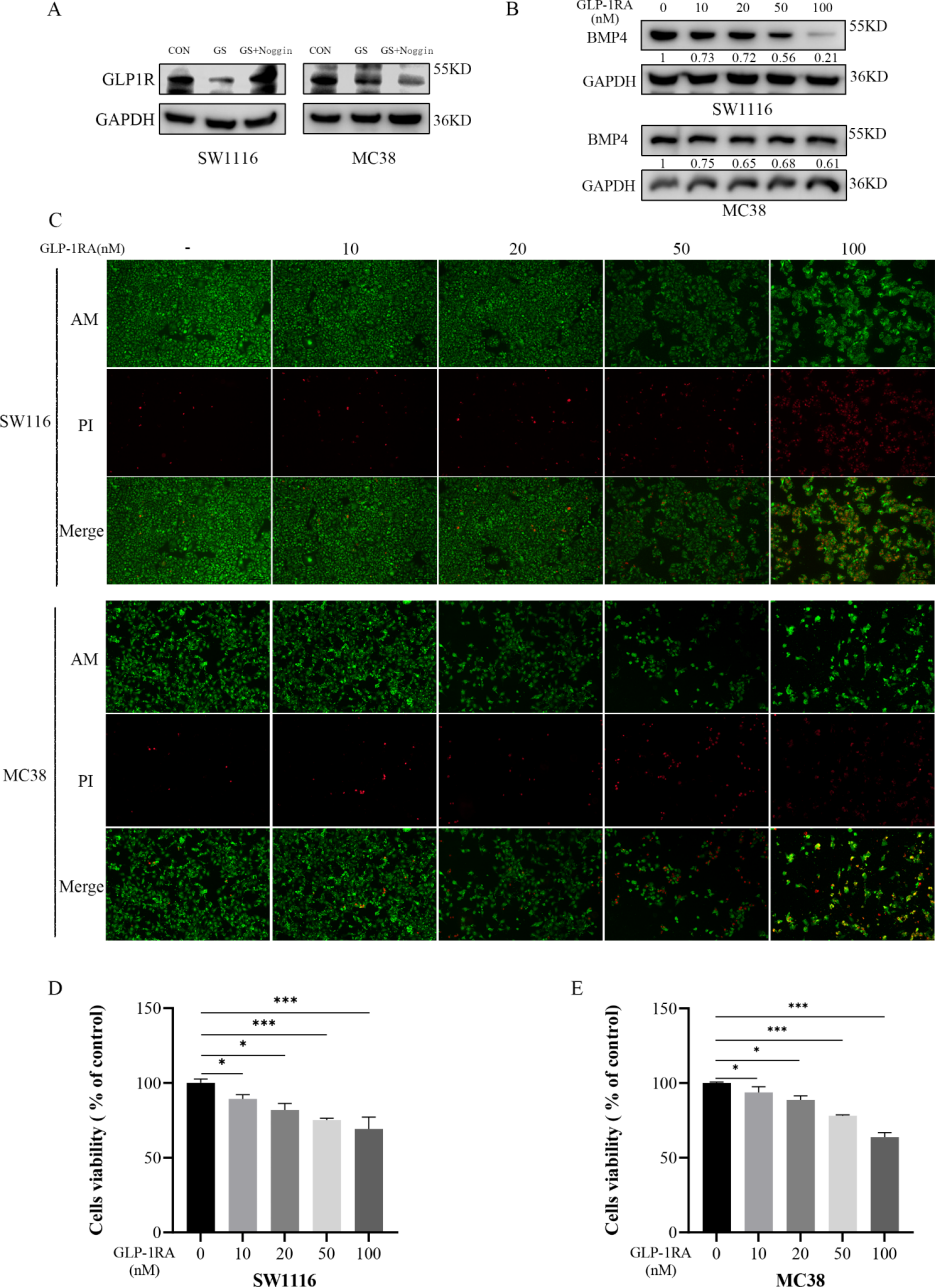
GLP-1RA diminished the proliferation of CRC. (**A**) The expression of GLP-1R was downregulated by high glucose presented by western blot (**B**) Western blotting analysis of the expression of BMP4 in SW1116 and MC38 treated with GLP-1RA, liraglutide, with different concentrations (0, 10, 20, 50, 100nM) for 48 h (**C**) The dead cells of SW1116 and MC38 intervened with GLP-1RA for 48 h were detected by Calcein‑AM/PI double staining (magnification, x100) (**D**, **E**) Cell viability of SW1116 and MC38 cells treated with GLP-1RA for 48 h were measured by CCK-8 kit, *P < 0.05, ***P < 0.001

Our previous study has illustrated that GLP-1 can decrease the BMP4 levels in the liver [25]. And, in this study, we first found a depressed expression of glucagon-like peptide-1 receptor (GLP-1R) in high-glucose treated CRC compared with the control group (Fig. 5A). Hence, we treated CRC with liraglutide injection, a GLP-1RA, to investigate whether GLP-1 affects the progression of CRC through medicating BMP4. As the results presented by western blotting, the expression of BMP4 decreased when SW1116 and MC38 cells were treated with GLP-1RA (Fig. 5B). Used the Calcein-AM/PI Double Stain Kit (40747ES76, Yeasen, China), live CRC cells were stained with red fluorescence and dead cells were stained with green fluorescence. With the increase of the concentration of GLP-1RA, the proportion of dead cells also increased (Fig. 5C). The Same result was proved by CCK-8 analysis, and we found that the GLP-1RA restrained the proliferation of CRC cells and demonstrated a dose-dependent growth inhibition (Fig. 5D, E). Hence, the assumption that GLP-1RA suppressed the proliferation of CRC cells was associated with decreasing BMP4 be proposed.

**Fig. 6:**
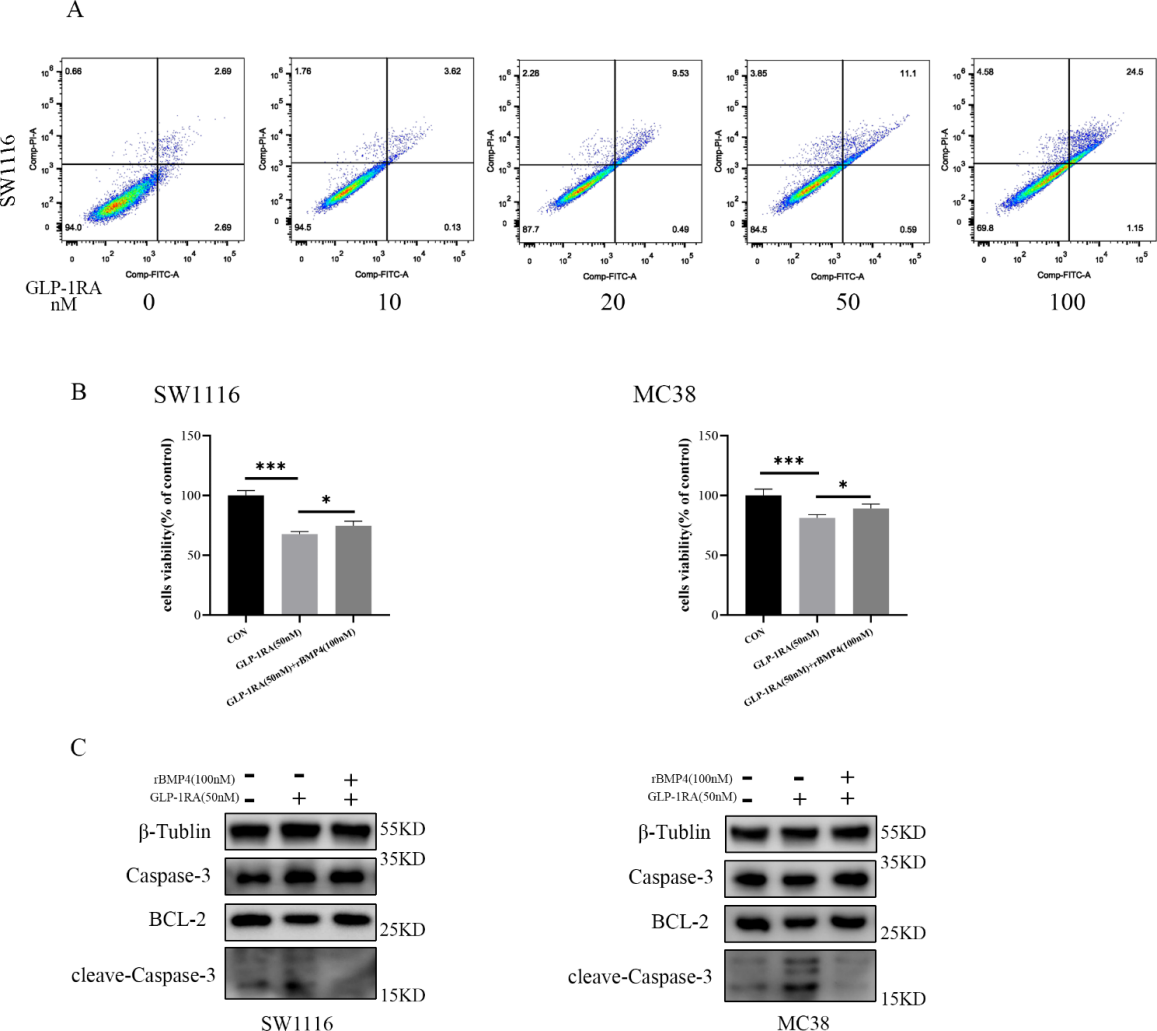
GLP-1RA promoted the apoptosis of CRC cells mediated by the dimmish of BMP4. (**A**) GLP-1RA promotes the apoptosis of SW1116 examined by flow cytometric analysis (**B**) Cell viability of SW1116 and MC38 cell lines cultivated by GLP-1RA and recombinant BMP4 for 48 h assessed by CCK-8kit (**C**) Western blot analysis of the expression of BCL-2, Caspase-3, and cleave-Caspase-3 in SW1116 and MC38 cell lines treated with GLP-1RA and recombinant BMP4.

Flow cytometric analysis of SW1116 cells after double staining with annexin V-FITC and propidium iodide fourthly showed the cytotoxicity of GLP-1RA (Fig. 6A). GLP-1RA mainly promoted the later stage apoptosis. We furtherly investigated whether BMP4 could offset the inhibition effect of GLP-1RA. And, cell viability assessed by CCK-8 kit, we found recombinant human BMP4(rBMP4) could partly reverse the suppressing effect induced by GLP-1RA (Fig. 6B). The expression of cleaved-caspase3 was upregulated, and BCL-2 was downregulated when treated with GLP-1RA, which could be reversed by rhBMP4 (Fig. 6C). These results showed that GLP-1RA, liraglutide, promoted the apoptosis of CRC cells mediated by the downregulation of BMP4.

### High glucose interaction with BMP4 regulated the metastasis of CRC in vivo

**Fig. 7:**
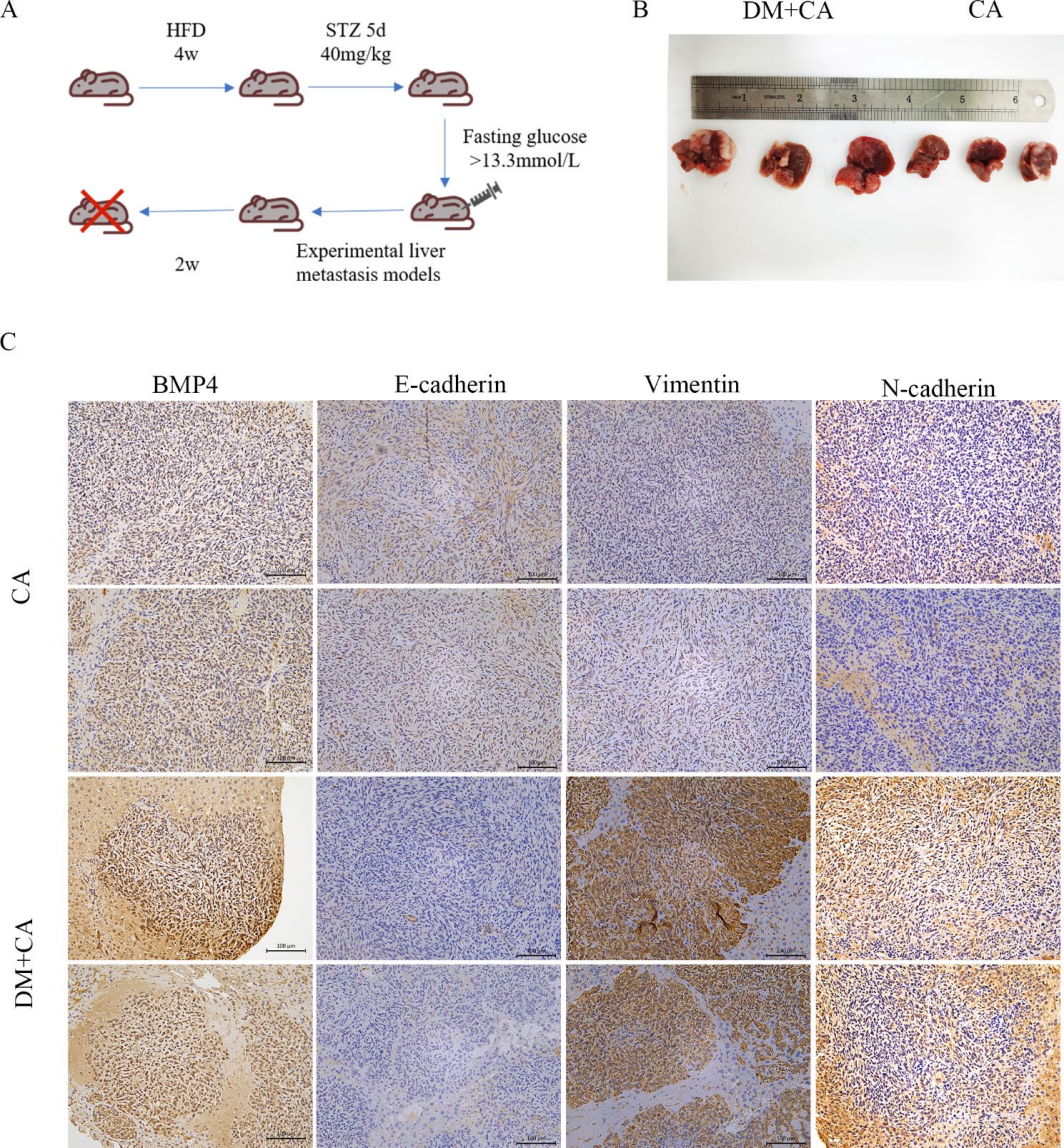
High glucose promoted the metastasis of CRC validated in vivo studies (**A**) Protocol for high-fat diet (HFD) and STZ-induced diabetes mouse models and liver metastasis model (**B**) Representative image of the liver of mice with diabetes (DM + CA group) and without diabetes (CA group) (**C**) BMP4 and E-cadherin, N-cadherin, Vimentin protein levels in formalin-fixed, paraffin-embedded tumors as detected by IHC.

After disclosing that high glucose induced IR could promote the metastasis of CRC through canonical BMP4-Smad pathway in vitro study, we further investigated the role of BMP4 in mediating the metastasis and EMT in CRC in vivo. We first established a diabetes mouse model and then performed the experimental liver metastasis model by injecting MC38 cells into the spleens of mice and the mice were sacrificed two weeks later [31](Fig. 7A). Compared with the mice without diabetes (CA group), the diabetes mice (DM + CA group) suffered more metastatic nodules (Fig. 7B). To further determine the role of BMP4 in EMT regulation in vivo, we evaluated the expression levels of EMT markers in the metastatic liver xenograft model. IHC pictures of tumor tissue sections from the flanks of the nude mice demonstrated that diabetes mice upregulated BMP4, Vimentin and N-cadherin expression but downregulated E-cadherin expression compared to the control group (Fig. 7C), revealing that IR may induce BMP4 to medicate the EMT of CRC.

## Discussion

The prevalence of DM has been increasing over the past decades, which diminishes the quality of patients and increases the medical care cost and social burden [36]. Epidemiological studies have shown that diabetes is associated with many kinds of cancer happening and cancer mortality [37–39]. Compared with patients without diabetes, diabetes patients have a significantly higher risk of CRC and a higher risk of early-onset CRC [40]. Besides, DM also increases the risk of BRAF-mutated tumors and the proliferation and metastasis capacity of tumors [41, 42]. Even for patients who accepted surgery treatment, diabetes also increases the occurrence of postoperative complications and poorly affects the outcomes of CRC patients [43]. The presence of DM is associated with the occurrence of CRC, and the presence of cancer conversely increases the risk of death in CRC patients with DM [4, 44, 45]. Hence, some indirect or direct relationships between CRC and DM may exist.

Until now, the detailed mechanism between DM and cancer has not been clarified clearly. And, the abnormal accumulated glucose and insulin are instrumental in the transformation and progression of cancer. Hyperglycemia and IR can induce oxidative stress, promote cytokines expression, and activate the inflammation pathway [46]. Hyperglycemia is associated with oxidative stress, further triggering DNA damage and facilitating carcinoma [47].IR induces the expression of IL-6, a pro-inflammatory, and promotes tumorigenesis [48]. A study also showed that DM and CRC share some common miRNAs, which may partly explain the mechanism relationship between DM and cancer [49]. Therefore, exploring the critical molecule that links DM and CRC is needed.

BMP4 is a member of the TGF-β superfamily, which plays a vital role in adipocyte differentiation and thermogenesis and regulates glucose homeostasis and insulin sensitivity [10]. BMP4 is increased in patients with metabolic disorders such as DM, obesity, and nonalcoholic fatty liver disease(NAFLD), and its level is negatively associated with insulin sensitivity [22, 50]. Besides, the upregulated BMP4 also has been reported to be associated with many cancers and negatively associated with overall survival [51, 52]. The aberrant activation of the Wnt/β-catenin pathway could increase the expression of BMP4, facilitating the invasiveness and tumorigenesis of CRC [15]. There also has study investigated the gene expression difference in CRC patients with or without KRAS mutation, and found that BMP4 elevated with the mutation of KRAS, which provided a novel treatment target for these patients [53]. However, no study has investigated the expression difference of BMP4 in CRC with or without T2DM and the underlying mechanism of BMP4 links to CRC and diabetes.

In this study, we demonstrated that high glucose induced IR could activate the expression of BMP4 in CRC cells and the tumor of CRC patients with DM. CRC cell lines treated with high glucose showed a higher capacity for proliferation and metastasis when compared with the control group. And, these promotion effects could be reduced by Noggin, the extracellular inhibitor of BMP4. BMP4 played its role mainly through the canonical pathway, which combined with BMPR and increased the affinity of intracellular receptor R-Smad proteins (Smads-1/5/8) binding to Smad4. Then the complex (Smad-4/R-Smads) is translocated into the nucleus and combined with DNA to regulate gene expression [54]. In our study, we found Smad1/5/8 was phosphorylated by high glucose and the marker of EMT was dysregulated in the high glucose group, the E-cadherin downregulated, and the N-cadherin, Vimentin, Snail upregulated. These results showed that high glucose induced IR mediated the metastasis of CRC through the BMP4-Smad-EMT pathway.

Metastasis is one of the properties of cancer, and the cancer cells shed from the primary tumor is a vital step in this progress. EMT, where cancerous epithelial cells lose their cell-to-cell contact and develop a more motile and less differentiated mesenchymal phenotype, has been reckoned as a crucial biological step driving tumor cell invasion and metastatic dissemination from primary tumors [34, 55]. The liver is recognized as the most common site of CRC metastasis because most of the intestinal mesenteric drainage enters the hepatic portal venous system. More than 50% of patients with CRC will develop metastatic disease to their liver throughout their life, ultimately resulting in death for more than two-thirds of these patients. The liver is the most common site of metastasis for colorectal cancer, and only 15–20% of patients with liver metastases are suitable candidates for surgical resection [56, 57]. Growing evidence proved that elevated expression of BMP4 is associated with the EMT of various epithelial malignant carcinomas [17]. And, the result of our study verified that BMP4 induced by high glucose has an effect on EMT that may be helpful in finding a novel therapeutic target for CRC patients with metastasis.

We have reported that GLP-1RA could suppress the expression of BMP4 in liver and adipose tissue. And, GLP-1RA is a commonly used second-line therapy in clinical to manage T2DM [58]. However, due to the concerns about safety for cancers, the clinical usage for CRC complicated by T2DM was relatively limited [58]. A cohort study reported that using GLP-1RA would not increase the risk of CRC [59]. In addition, there are emerging reports on the anti-cancer effects of GLP-1RA, but the mechanisms require further elucidation. GLP-1RA attenuates the proliferation of pancreatic cancer by inhibiting PI3K/Akt and the nuclear factor-kappa B(NF-kB) signaling pathway [60, 61]. Decreased expression of GLP-1R has been reported in pancreatic β cells of diabetes and pancreatic cancer tissues [62, 63]. Our study also found that the expression of GLP-1R was downregulated in CRC cells treated with high glucose. Hence, we investigated whether GLP-1RA mediates the growth of CRC cells through BMP4. And, we found that BMP4 was decreased by GLP-1RA, which promotes cleaved-caspase3 generation and BCL-2 downregulation and suppresses the growth of CRC cells. These results indicated that BMP4 mediated by GLP-1RA induces the apoptosis of CRC cells and provided new proof for the safe use of GLP-1RA in CRC patients.

Our study focused on the effect and underlying mechanism of DM on CRC and our findings indicated opportunities for BMP4 as a novel therapeutic target to reduce metastatic burden in patients and provided evidence that GLP-1RA could be safely used for CRC patients with comorbidity of diabetes.

## Conclusions

The upregulation of BMP4 induced by high glucose promotes the proliferation and metastasis of CRC cells. High glucose induced IR promotes the EMT of CRC mainly through the upregulation of BMP4, which activates the canonical pathway. Additionally, GLP-1RA has a strong inhibition effect on the growth of CRC cells by inhibiting the expression of BMP4. Therefore, BMP4 may represent a potential treatment target, and further studies are required to elucidate the therapeutic potential in colorectal cancer patients with T2DM.

## Electronic supplementary material

Below is the link to the electronic supplementary material.

Supplementary Material 1

## Data Availability

The datasets used and/or analyzed during the current study are available from the corresponding author on reasonable request.

## List of abbreviations

BMP4: Bone morphogenetic protein 4
T2DM: Type 2 diabetes mellitus
CRC: Colorectal cancer
GLP-1RA: Glucagon-like peptide-1 receptor agonist
IR: Insulin resistance
EMT: Epithelial-mesenchymal transition
TGF-β: Transforming growth factor-β
Smad: Signaling mothers against decapentaplegic
ERK: Extracellular signal-regulated protein kinase
PI3K: Phosphoinositide-3-kinase
OGTT: Oral glucose tolerance test
BMI: Body mass index
TNM: Tumor node metastasis
FBS: Fetal bovine serum
IHC: Immunohistochemistry
HFD: High-fat diet
STZ: Streptozotocin
OS: Overall survival
DFS: Diseases free survival
NAFLD: Nonalcoholic fatty liver disease
NF-κB: Nuclear factor kappa-B
