# Supplementary material for: High glucose promotes the progression of colorectal cancer by activating the BMP4 signaling and inhibited by glucagon-like peptide-1 receptor agonist
Source: BMC Cancer. 2023 Jun 27;23:594. doi: 10.1186/s12885-023-11077-w (PMC10304216; doi:10.1186/s12885-023-11077-w)

High glucose promotes the progression of colorectal cancer by activating the BMP4 signaling and inhibited by glucagon-like peptide-1 receptor agonist

Bingwei Ma[1]#, Xingchun Wang[2,3]#, Hui Ren[4], Yingying Li[4], Haijiao Zhang[5], Muqing Yang[6] and Jiyu Li[7]*

1 Colorectal Cancer Central, Shanghai Tenth People's Hospital, Tongji University, 301 Middle Yanchang Road, Shanghai, 200072, China

2 Department of Endocrinology and Metabolism, Shanghai Tenth People's Hospital, Tongji University, 301 Middle Yanchang Road, Shanghai, 200072, China

3 Thyroid Research Center of Shanghai, Shanghai Tenth People's Hospital, 301 Middle Yanchang Road, Shanghai, 200072, China

4 School of Pharmacy, East China University of Science and Technology, 130 Meilong Road, Shanghai, 200237, China

5 Department of Gastrointestinal Surgery, Huadong Hospital affiliated with Fudan University, 221 West Yanan Road, Shanghai, 200040, China

6 Department of General Surgery, Tenth People's Hospital of Tongji University, 301 Middle Yanchang Road, Shanghai, 200072, China

7 Geriatric Cancer Center, Huadong Hospital Affiliated to Fudan University, 221 West Yanan Road, Shanghai, 200040, China

#These authors have contributed equally to this work

*Corresponding author. Tel.: +86-21-66302531 Fax: +86-21- 66303046

E-mail addresses: lijiyu@fudan.edu.cn

Full-length gels and blots

Figure 2B BMP4

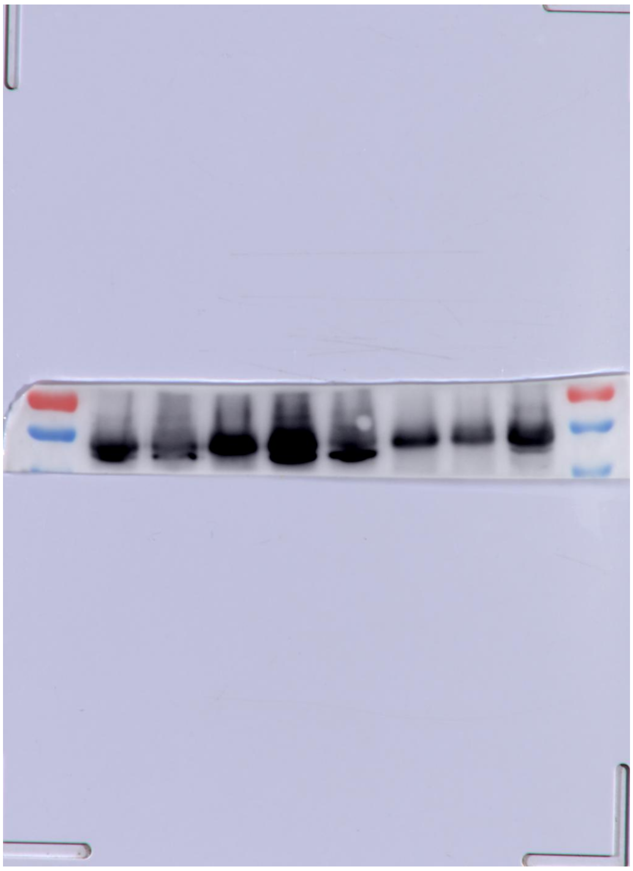

Figure 2B GAPDH

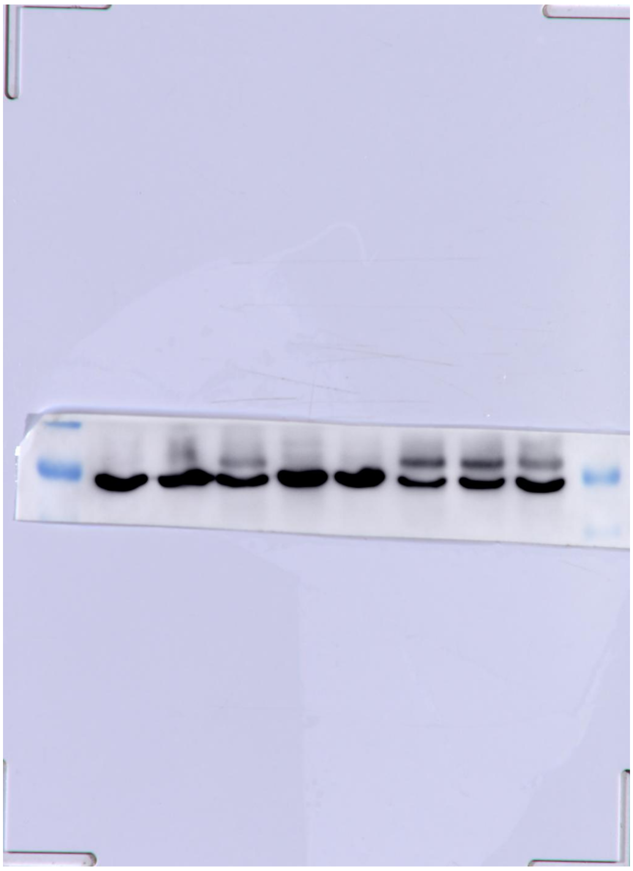

Figure 3C BMP4

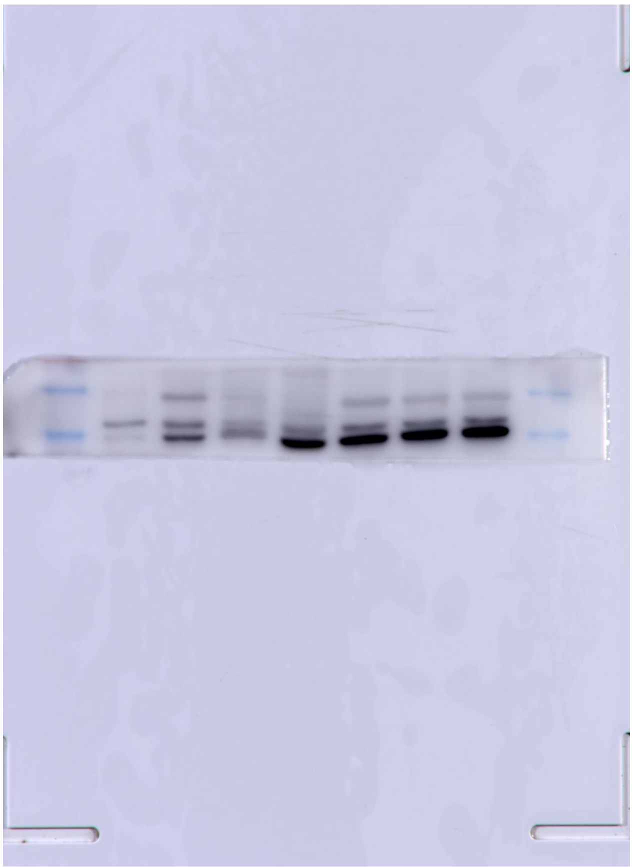

Figure 3C GAPDH

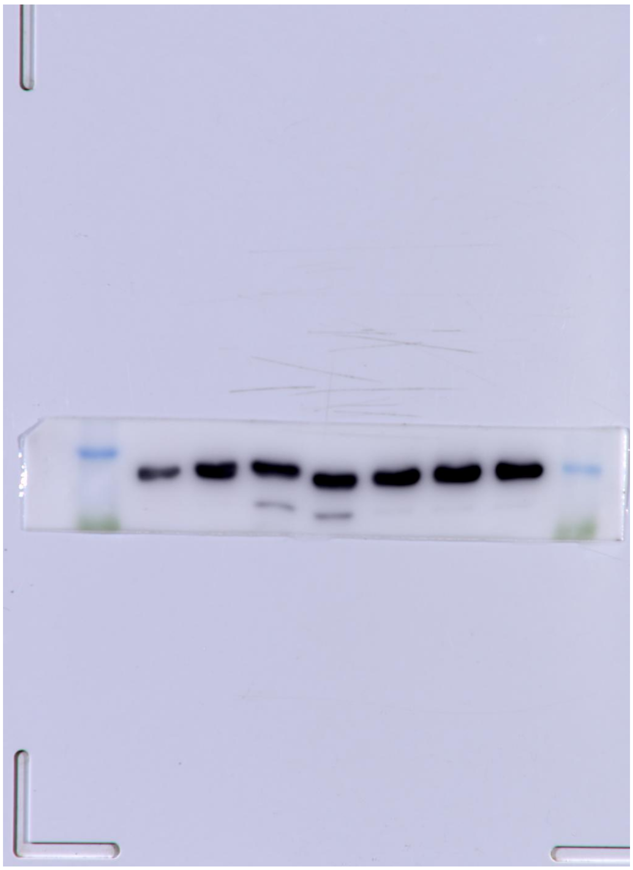

Figure 3D MC38 BMP4

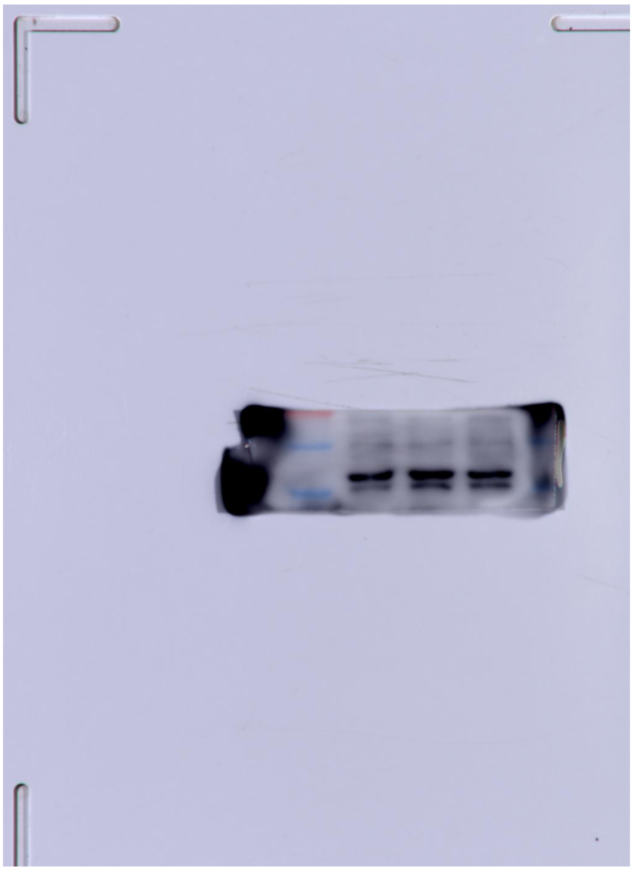

Figure 3D MC38 GAPDH

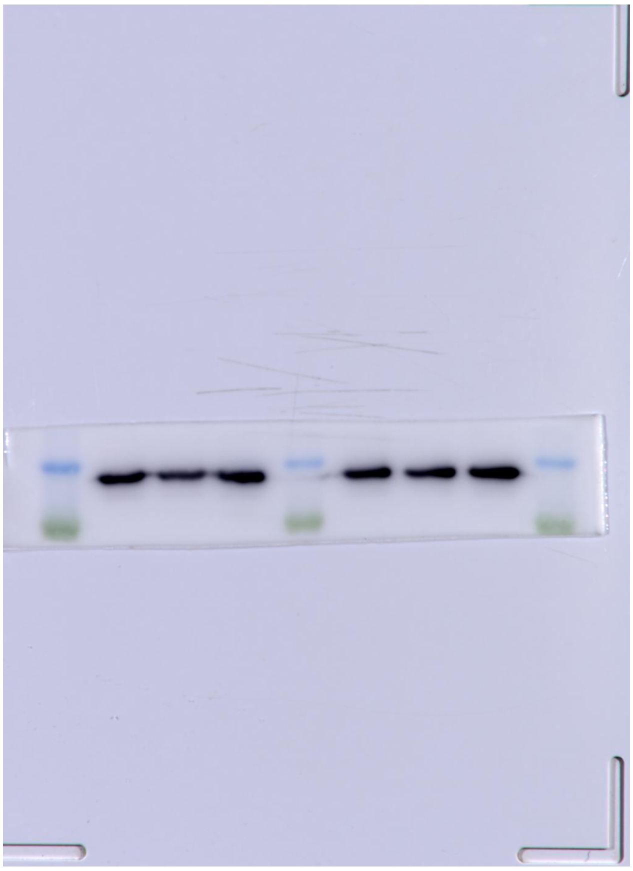

Figure 3D SW1116 BMP4

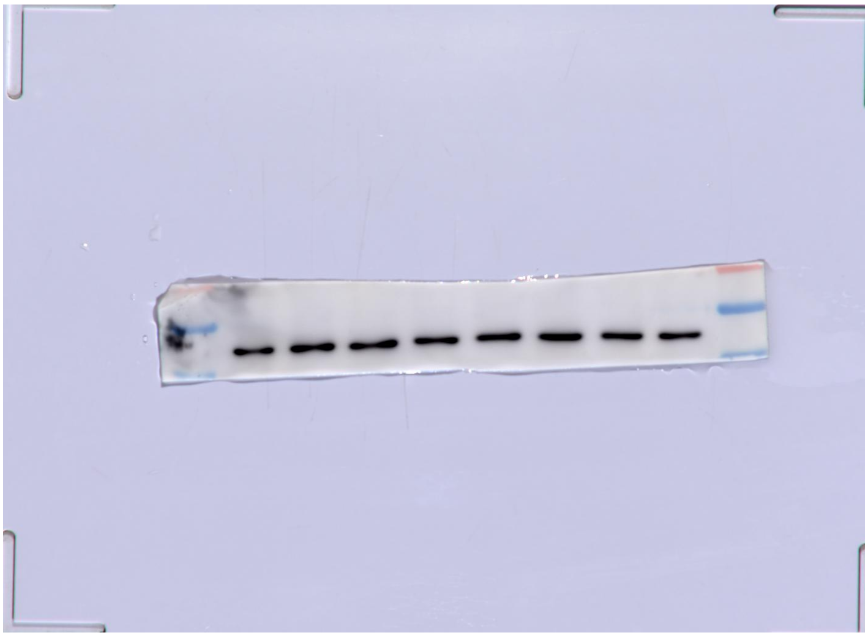

Figure 3D SW1116 GAPDH

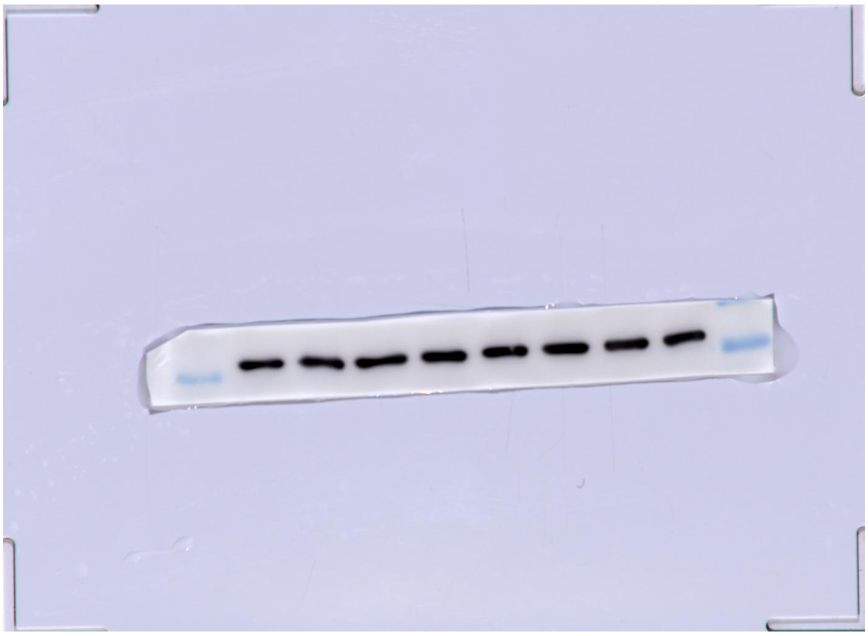

Figure 3D SW480 BMP4

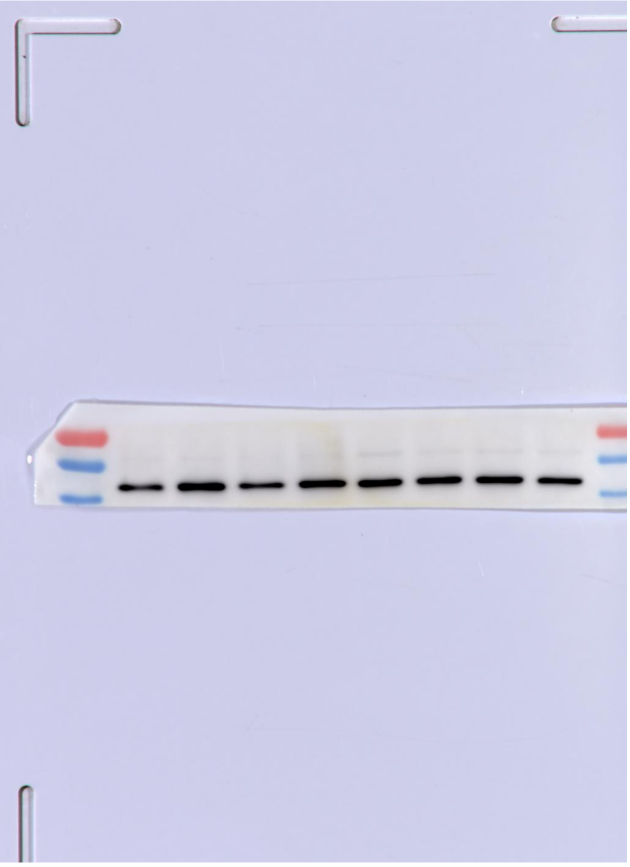

Figure 3D SW480 GAPDH

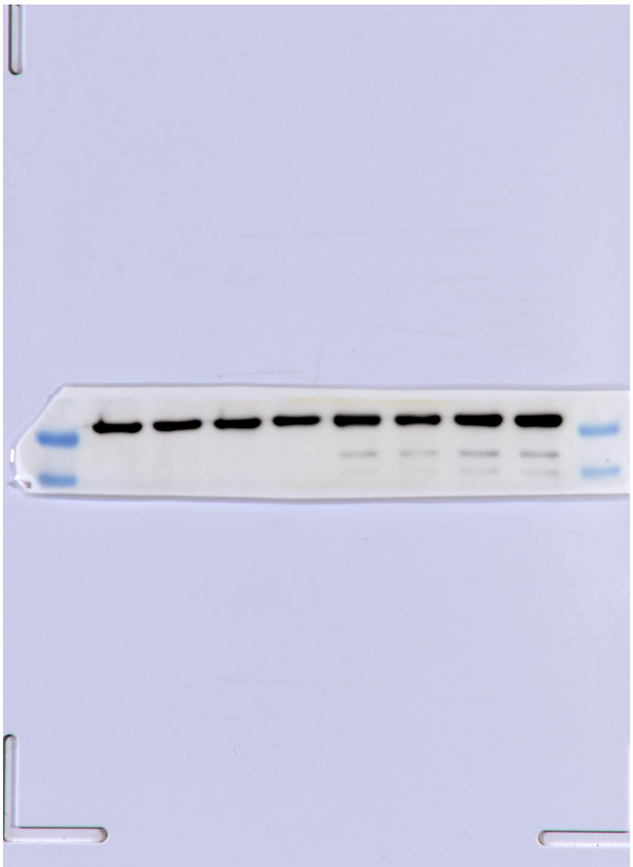

Figure 4A MC38 E-cadherin

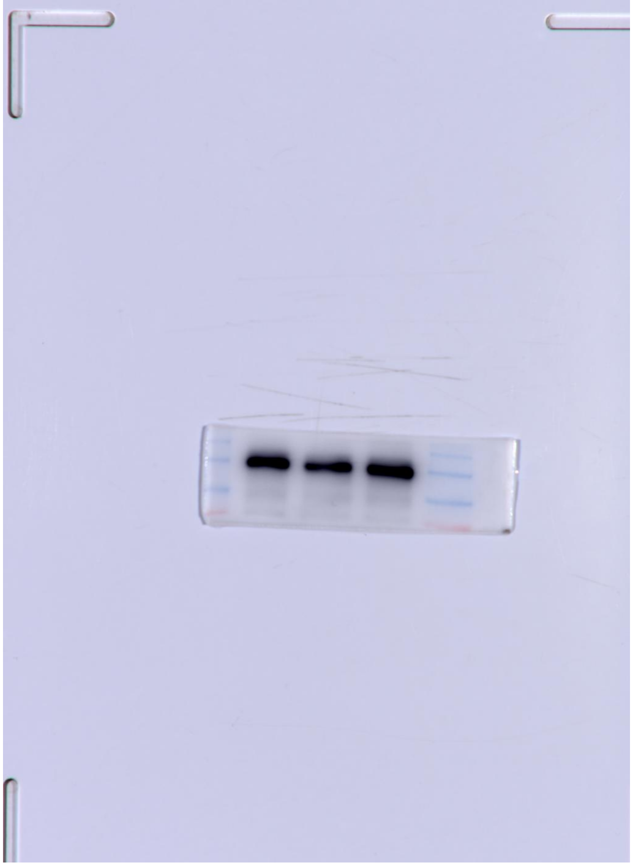

Figure 4A MC38 GAPDH

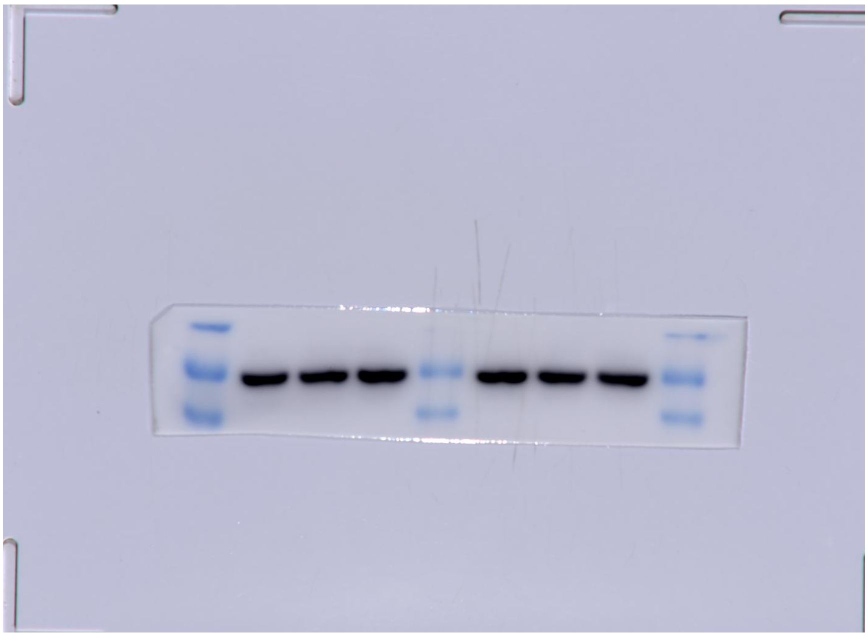

Figure 4A MC38 N-cadherin

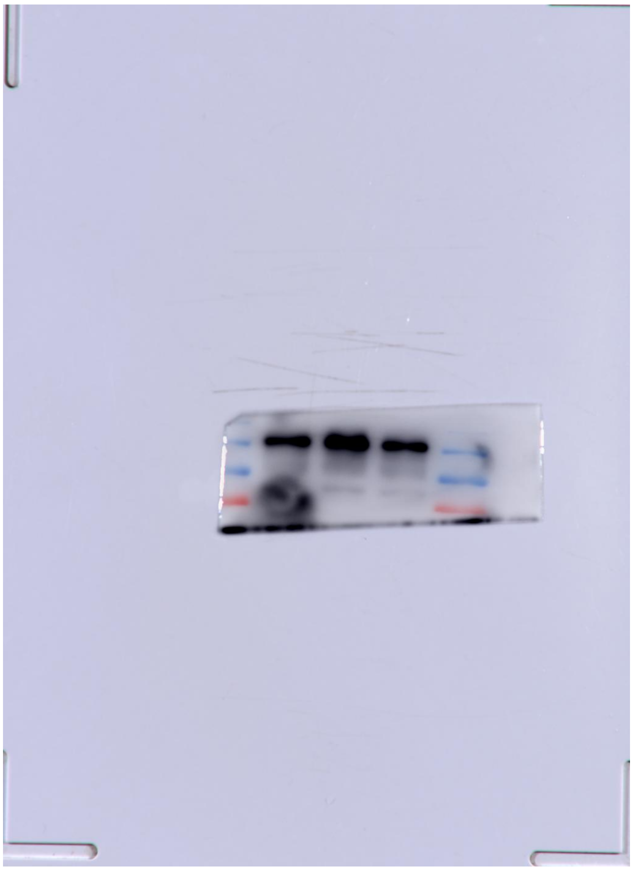

Figure 4A MC38 Snail

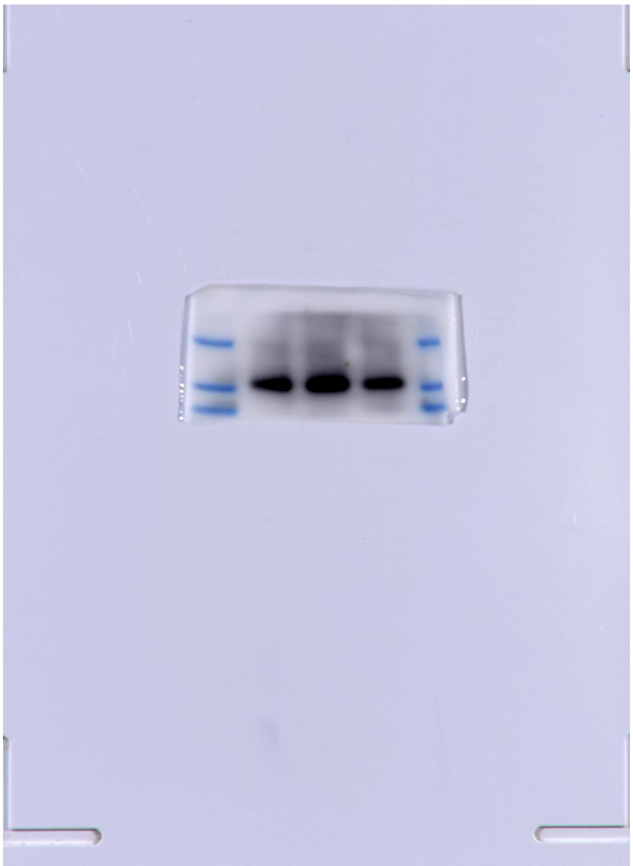

Figure 4A MC38 Vimentin

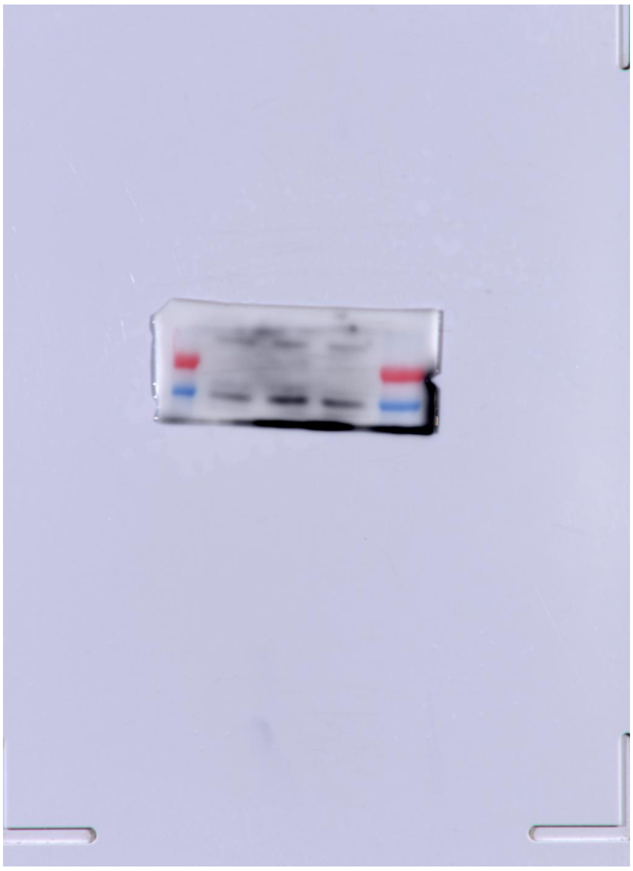

Figure 4A SW1116 E-cadherin

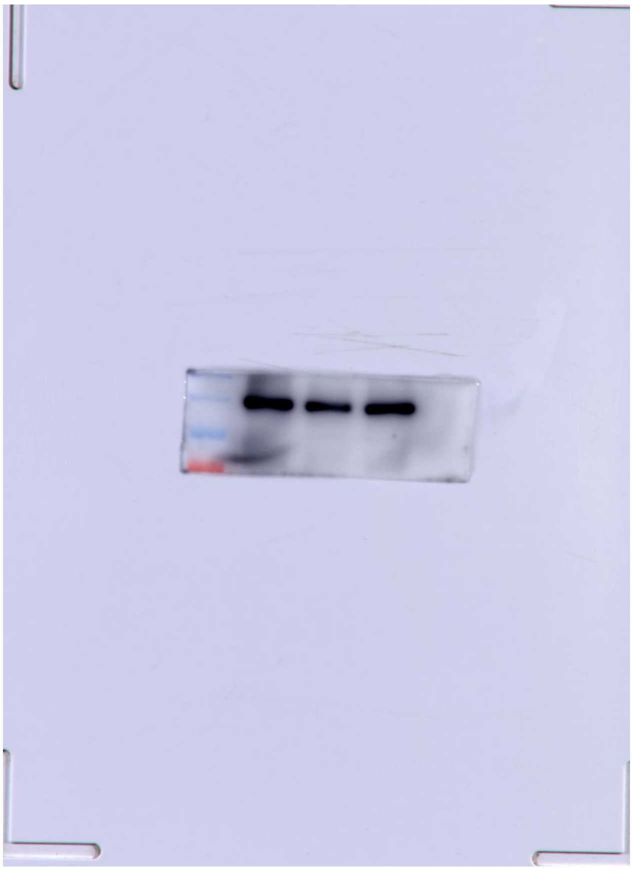

Figure 4A SW1116 GAPDH

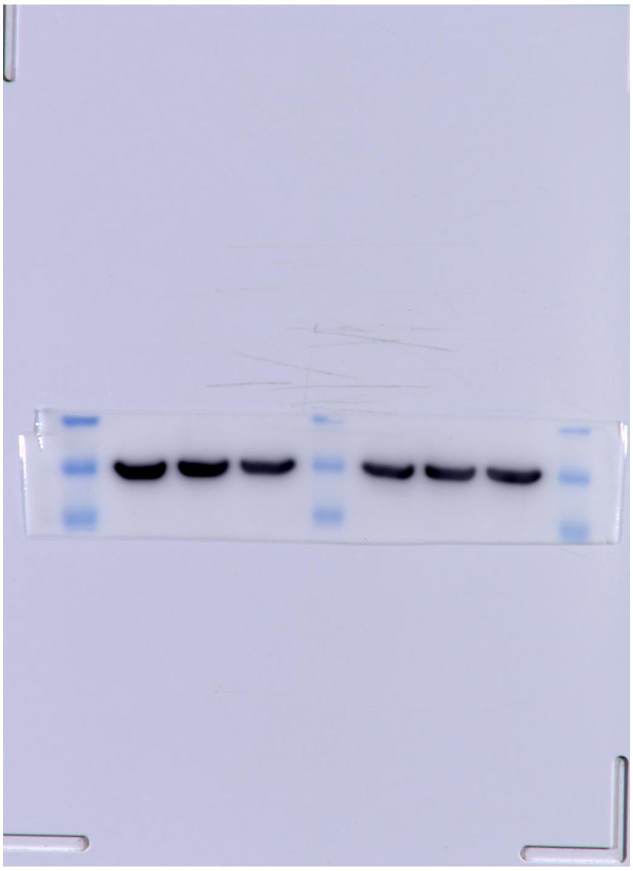

Figure 4A SW1116 N-cadherin

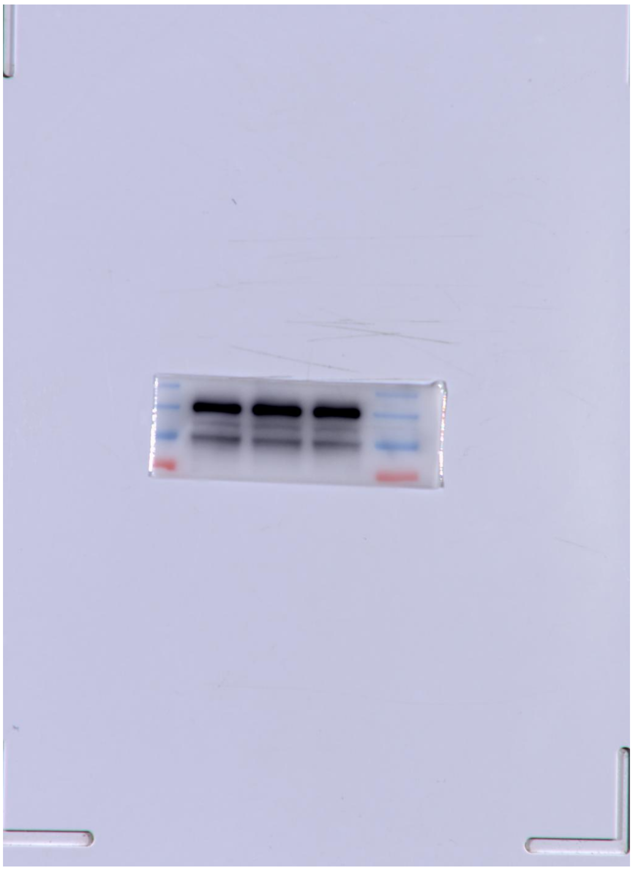

Figure 4A SW1116 Snail

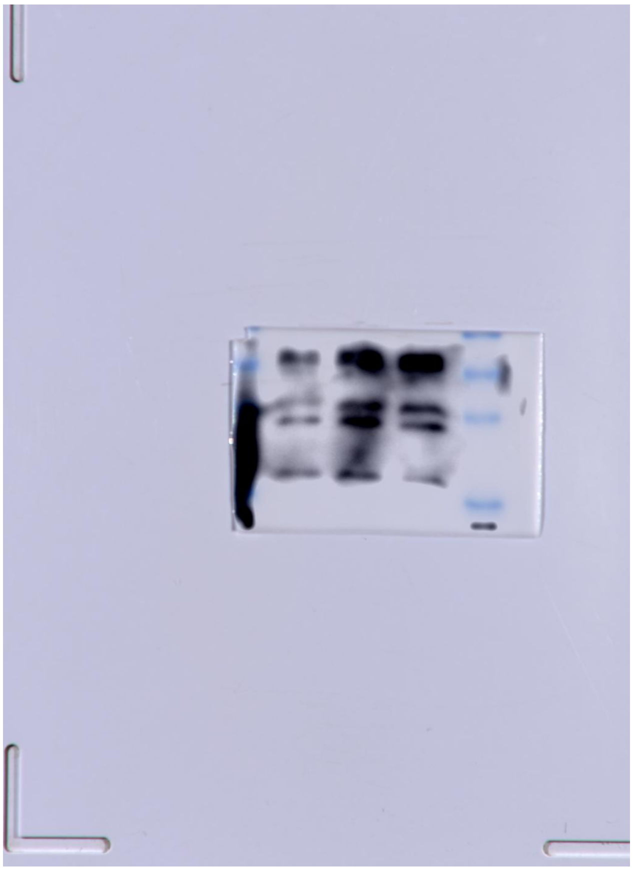

Figure 4A SW1116 Vimentin

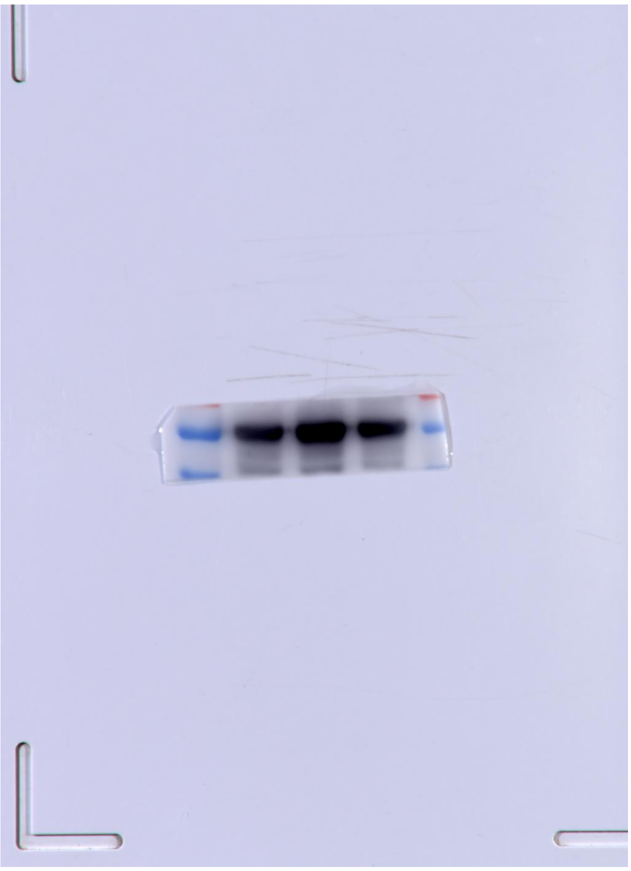

Figure 4B MC38 GAPDH

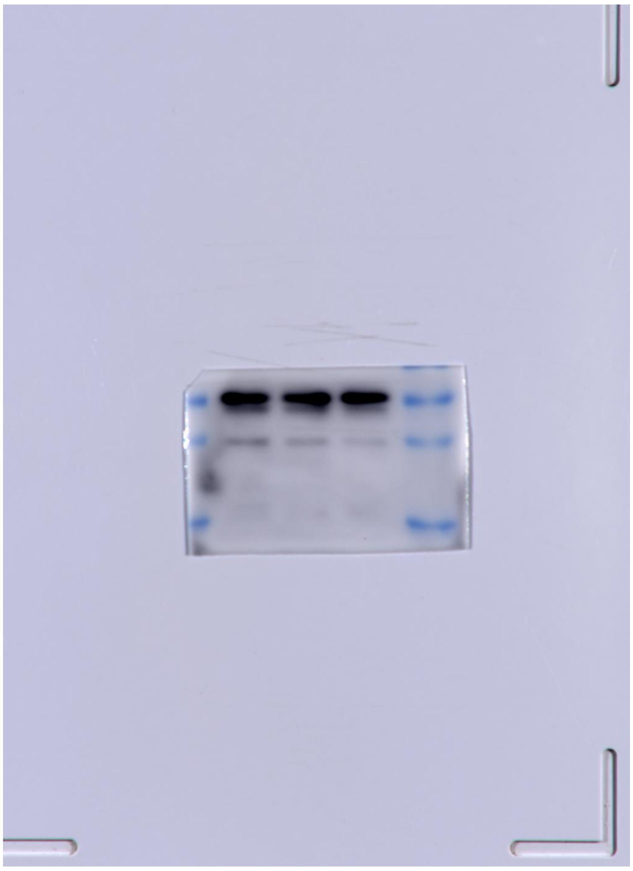

Figure 4B MC38 pSmad1/5/8

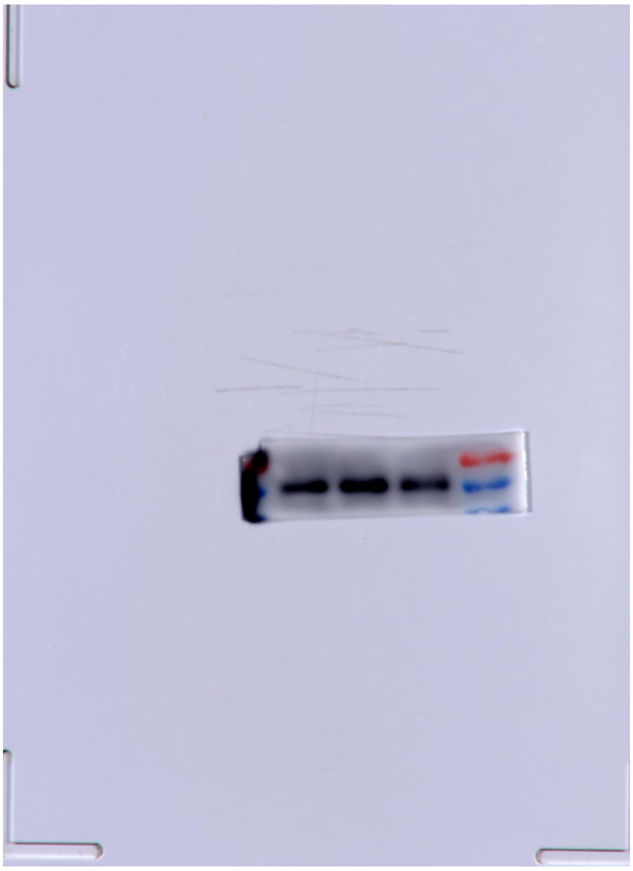

Figure 4B MC38 Smad1/5/8

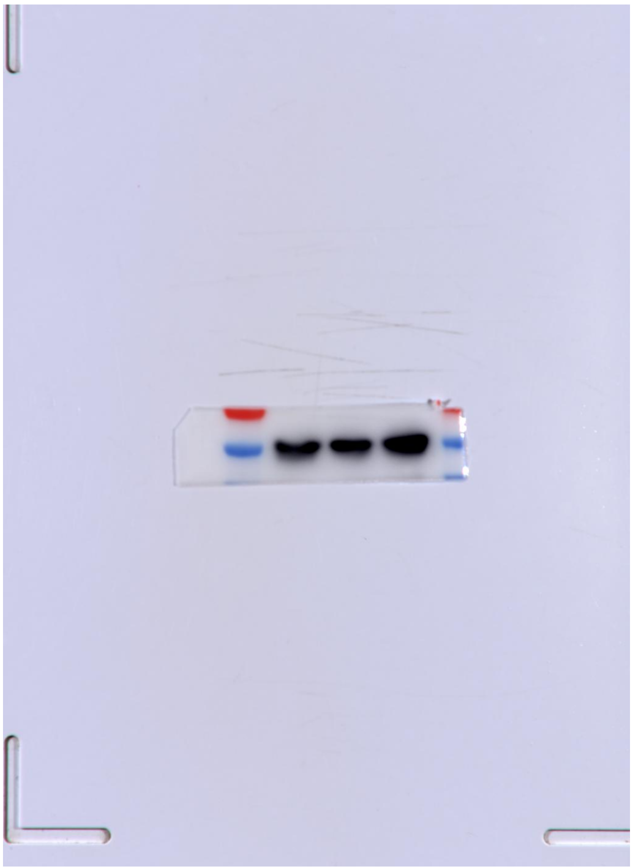

Figure 4B SW1116 GAPDH

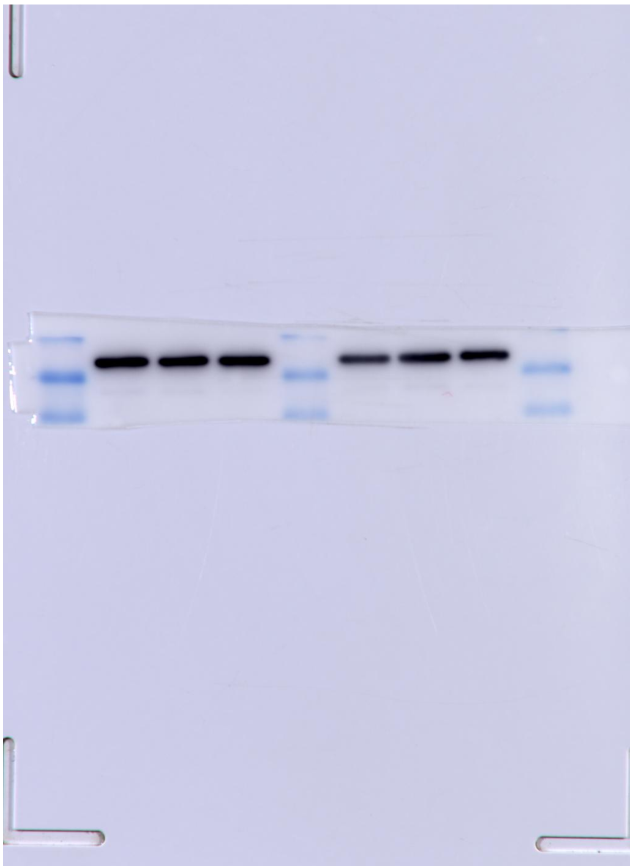

Figure 4B SW1116 pSmad1/5/8

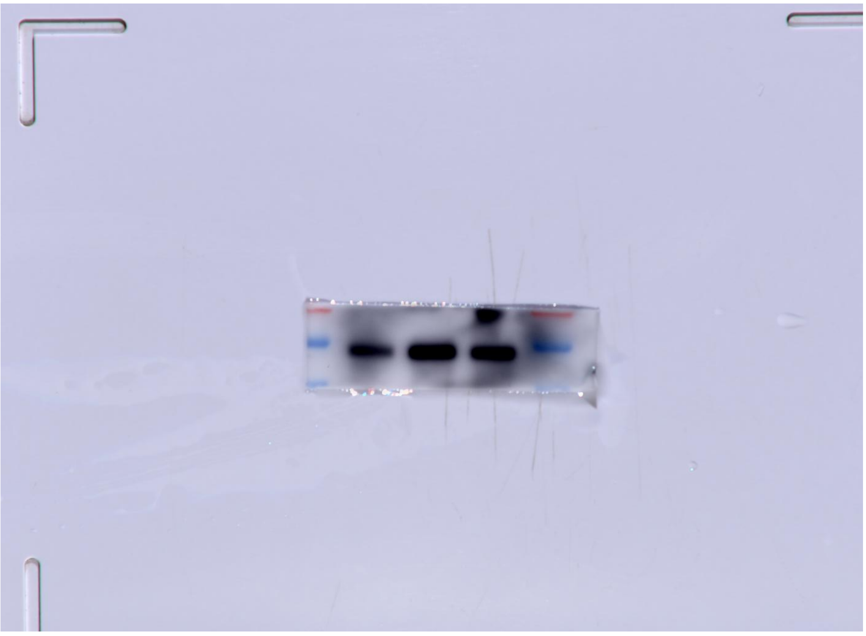

Figure 4B SW1116 Smad1/5/8

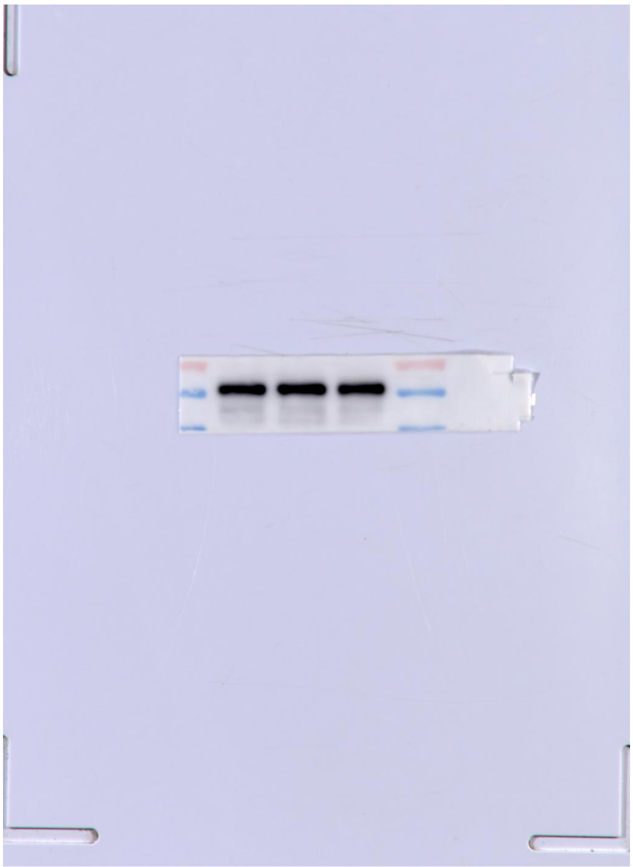

Figure 4C BMP4

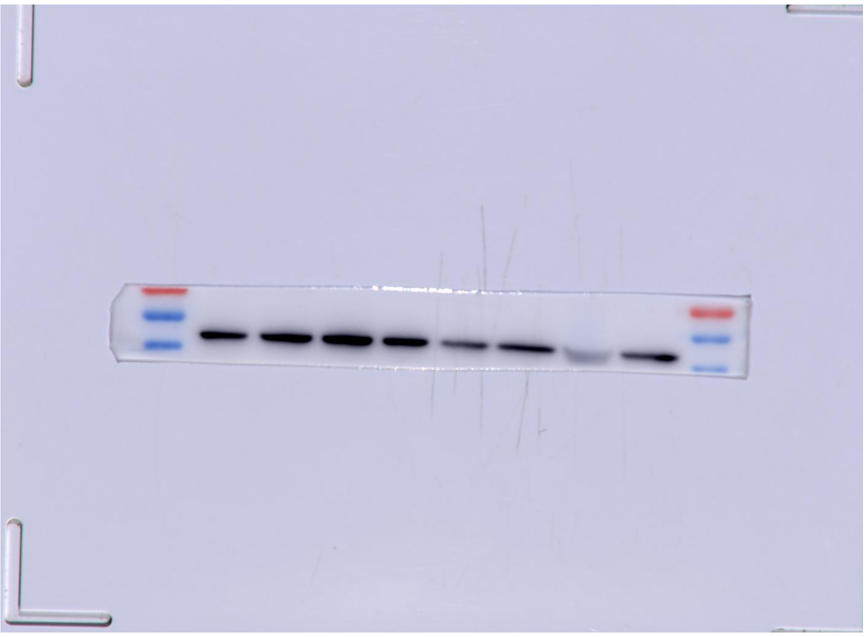

Figure 4C GAPDH

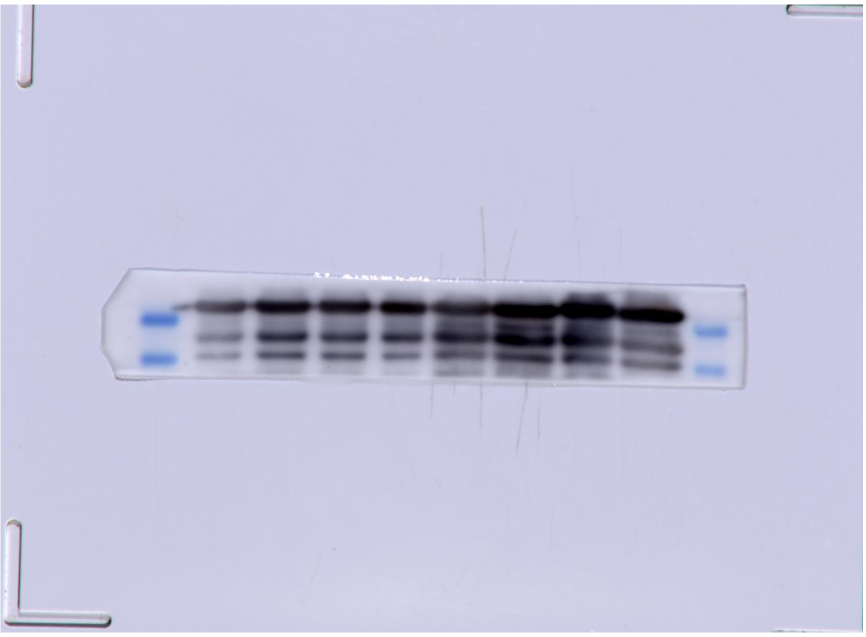

Figure 4D E-cadherin

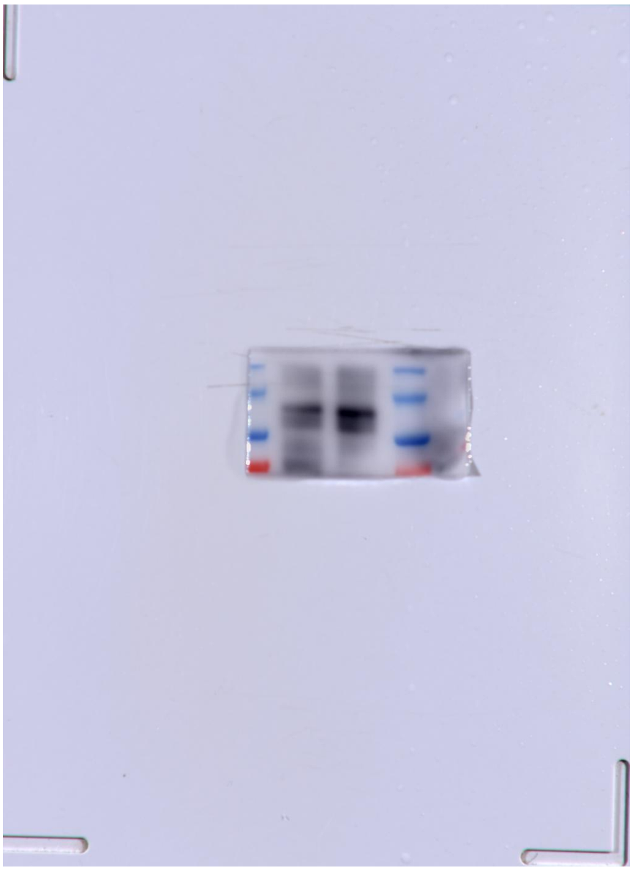

Figure 4D GAPDH

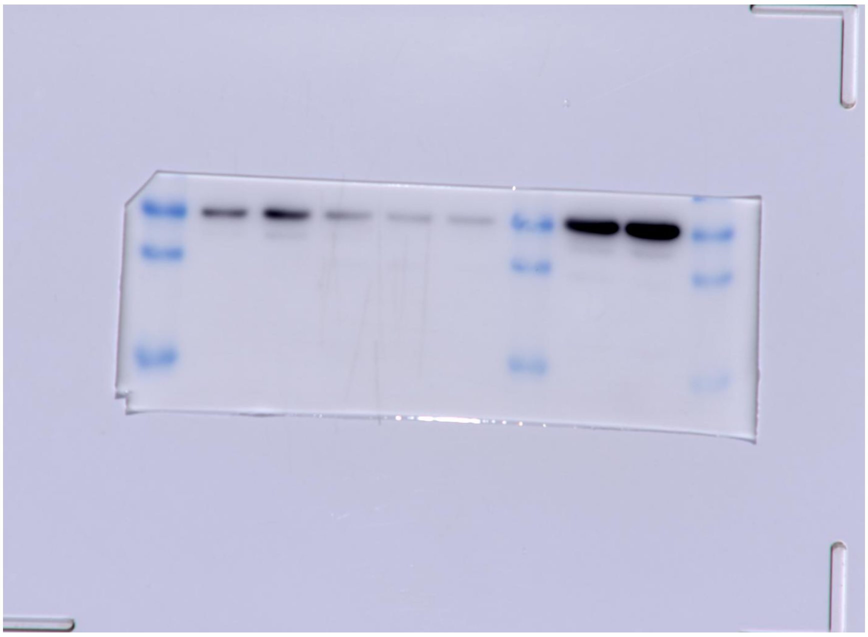

Figure 4D N-cadherin

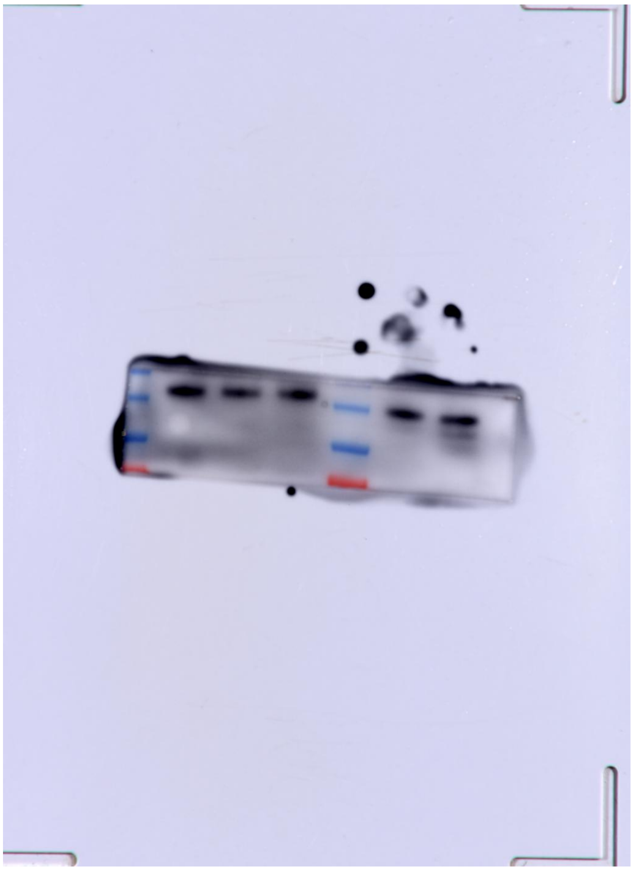

Figure 4D pSmad1/5/8

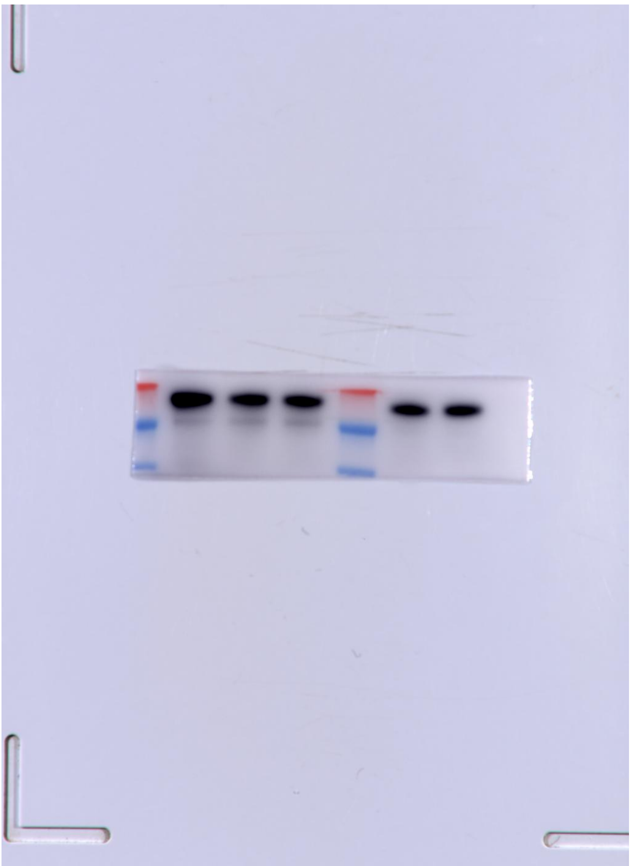

Figure 4D Vimentin

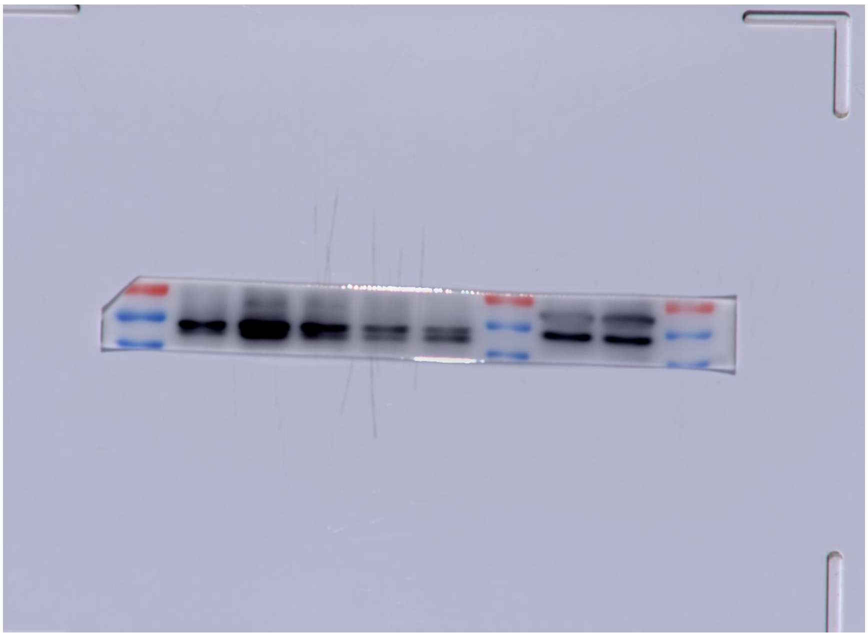

Figure 4D BMP4

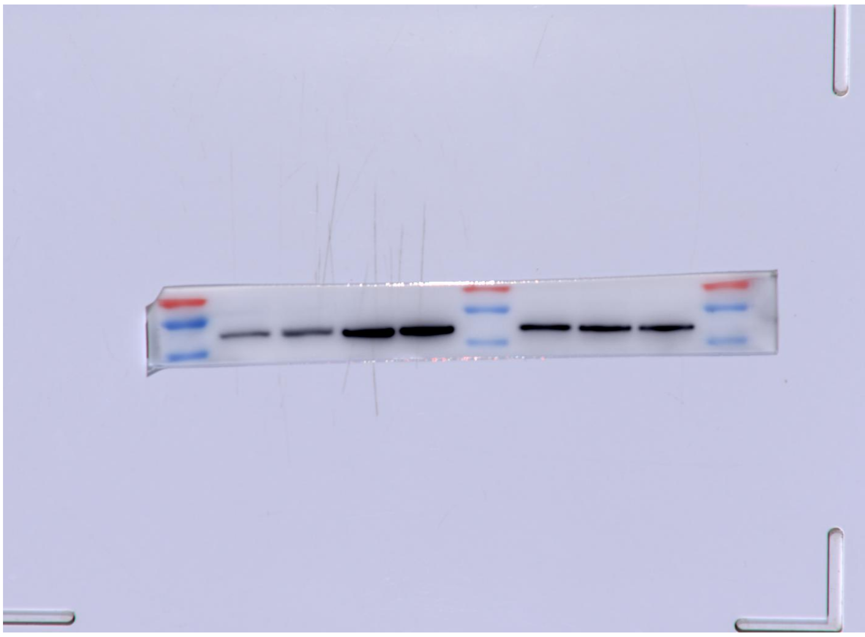

Figure 5A MC38 GAPDH

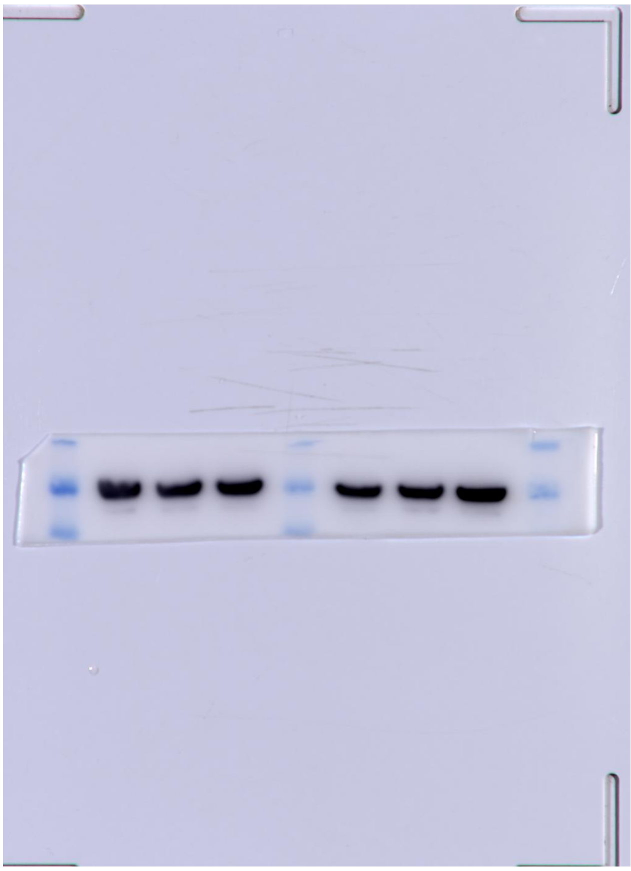

Figure 5A MC38 GLP-1R

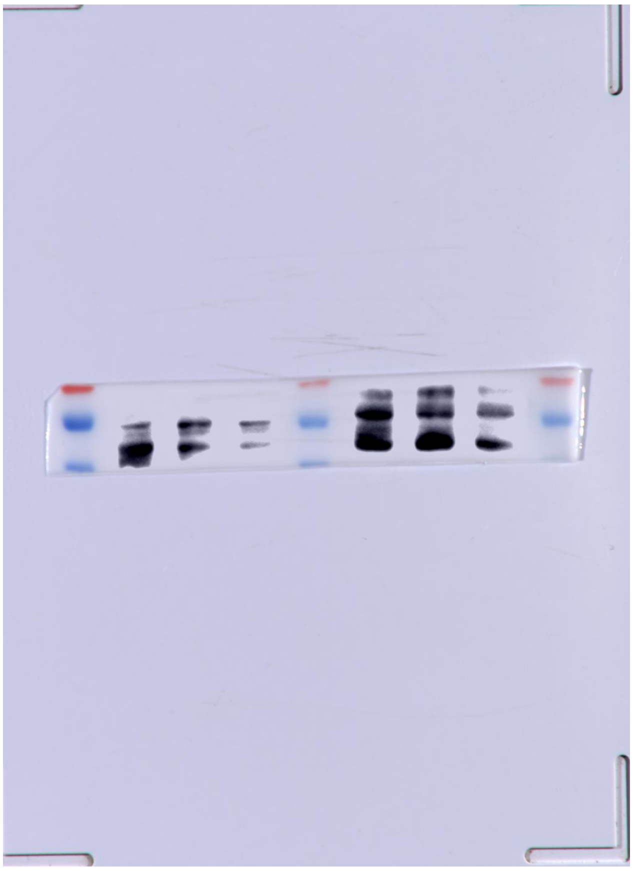

Figure 5A SW1116 GAPDH

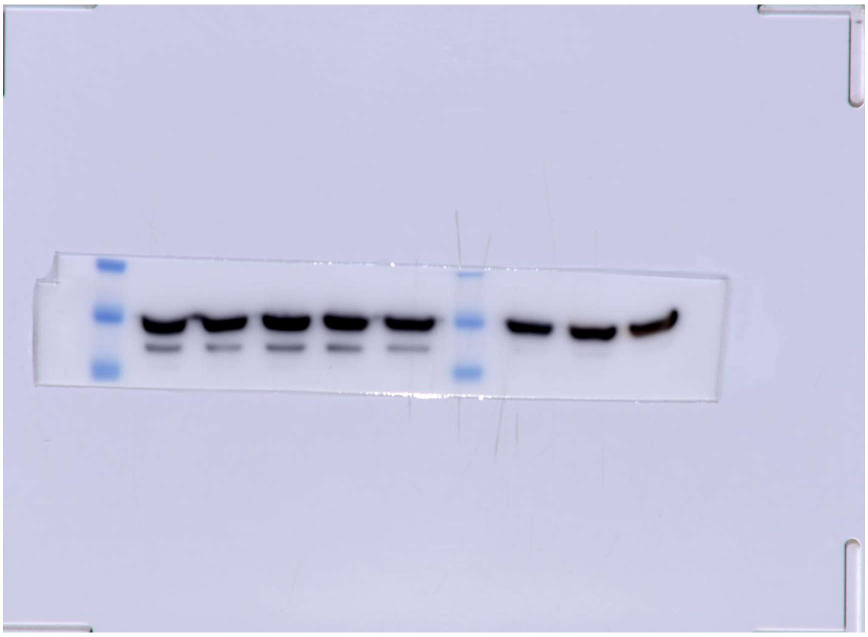

Figure 5A SW1116 GLP-1R

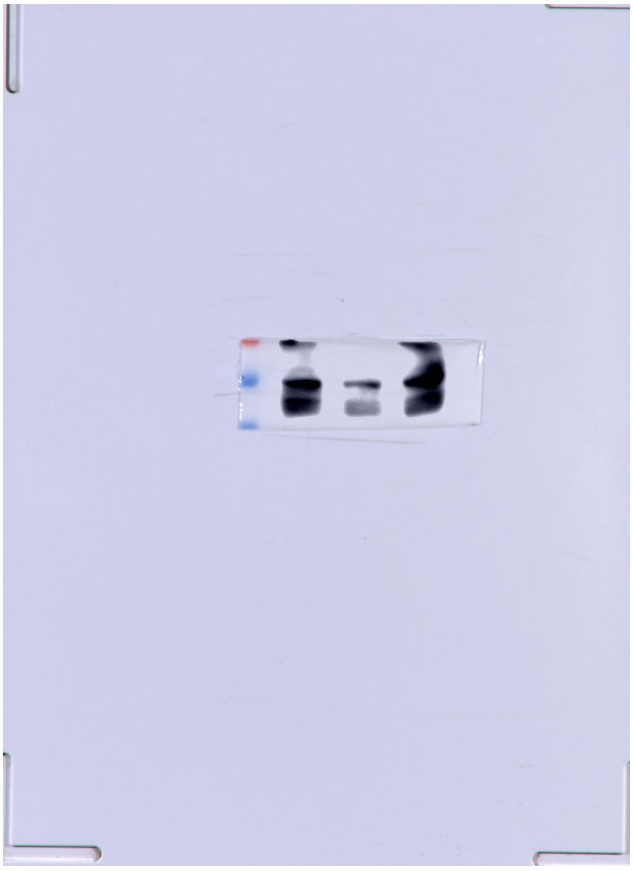

Figure 5B MC38 BMP4

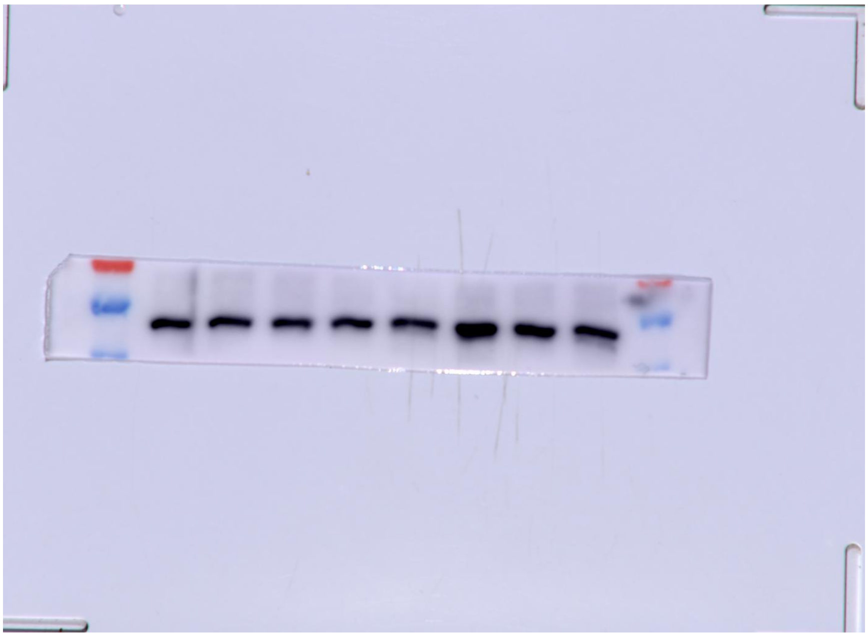

Figure 5B MC38 GAPDH

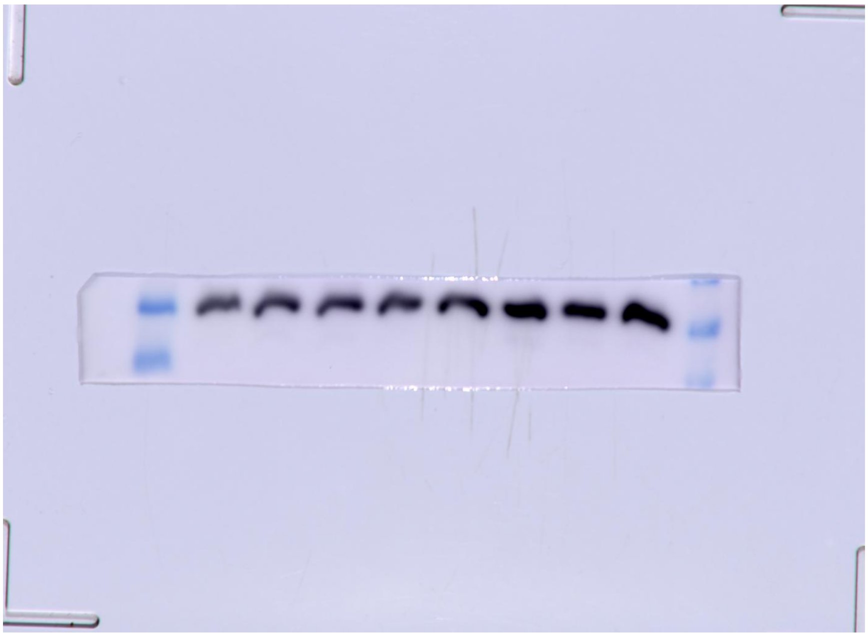

Figure 5B SW1116 BMP4

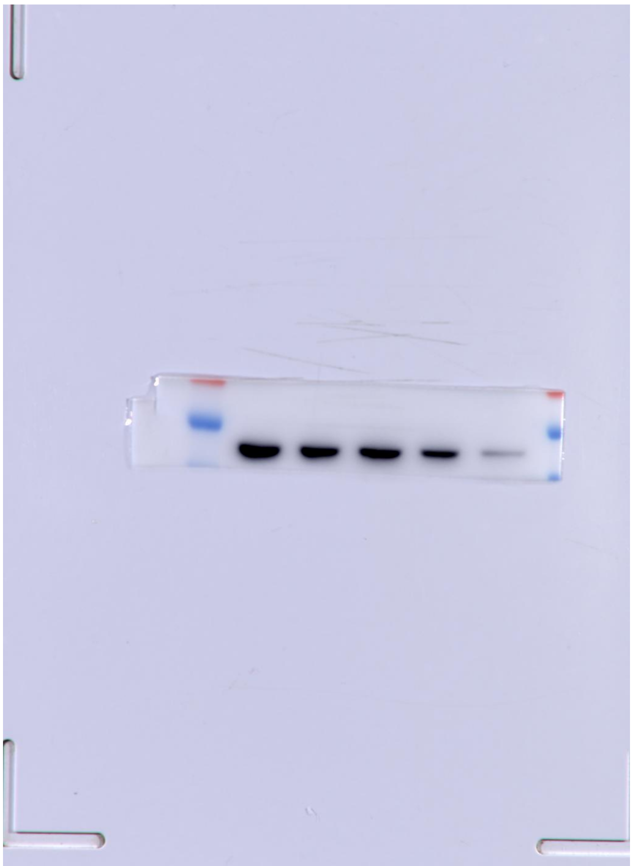

Figure 5B SW1116 GAPDH

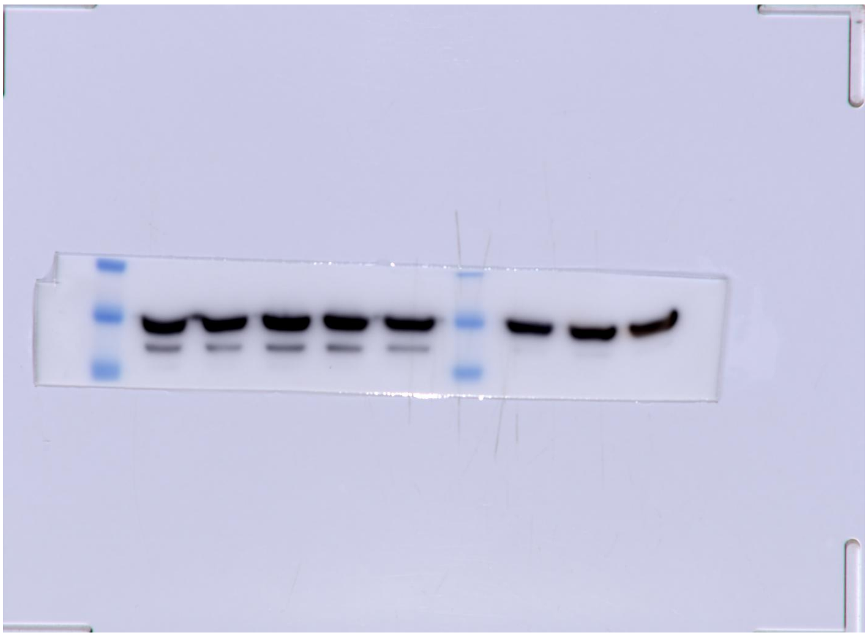

Figure 6C MC38 BCL2

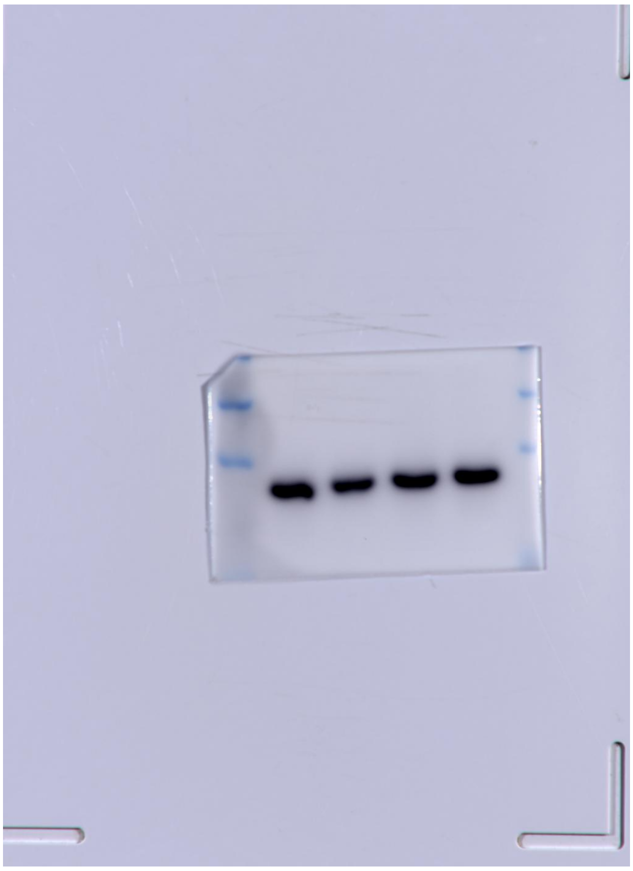

Figure 6C MC38 Caspase3

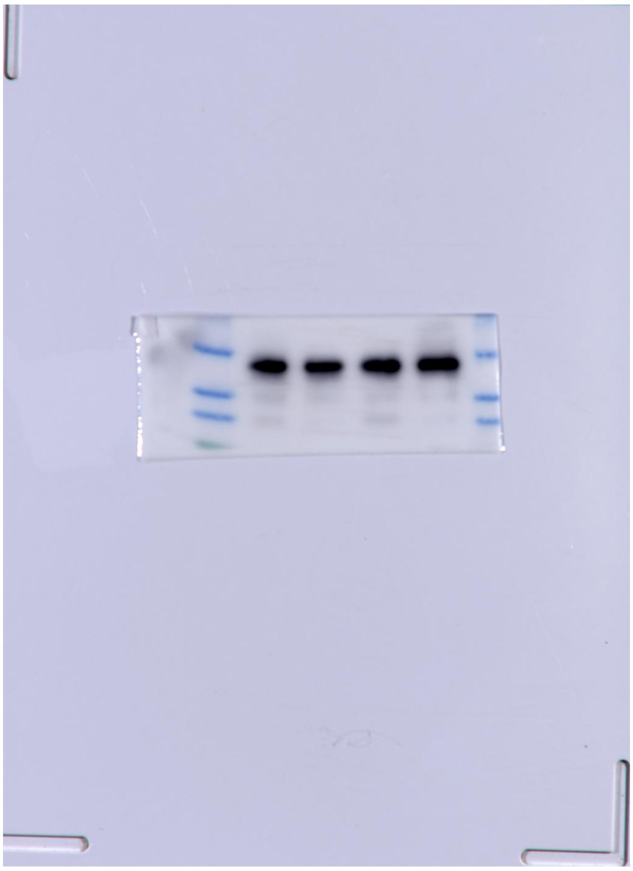

Figure 6C MC38 cleave-Caspase3

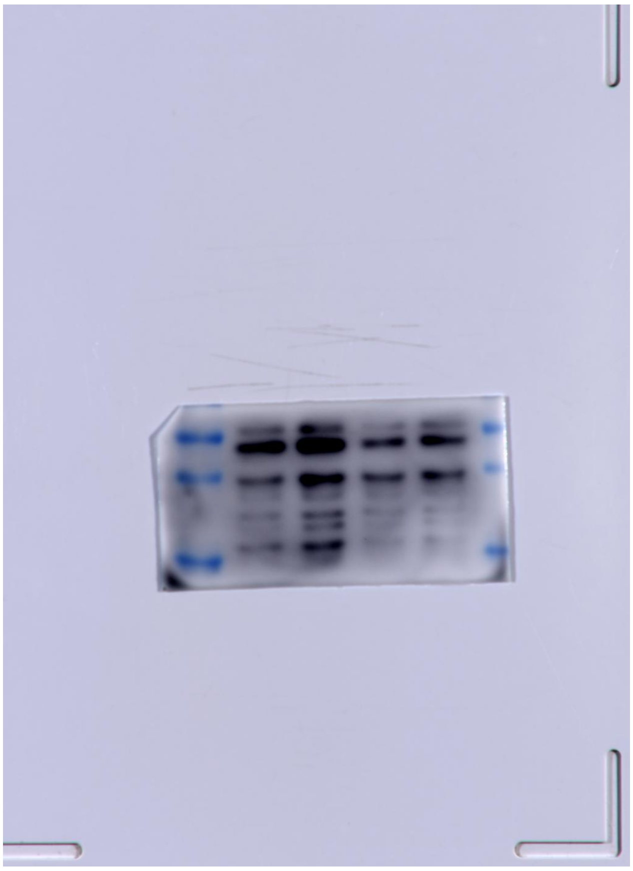

Figure 6C MC38 β-Tublin

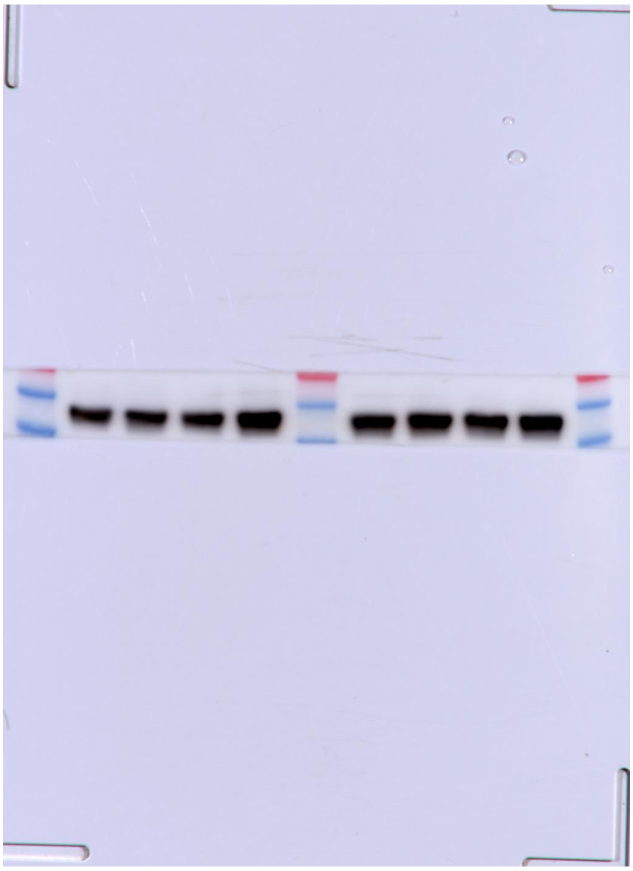

Figure 6C SW1116 BCL-2

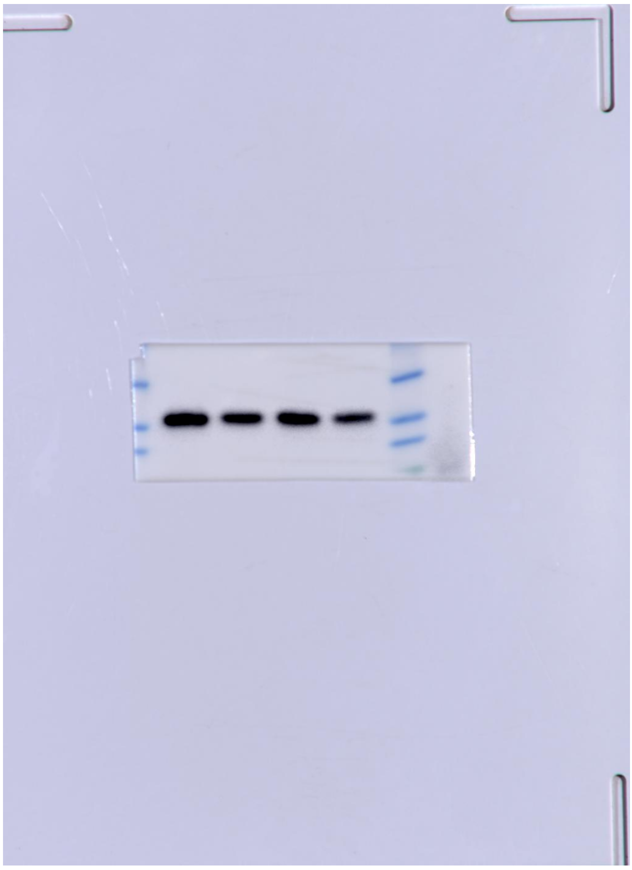

Figure 6C SW1116 Caspase3

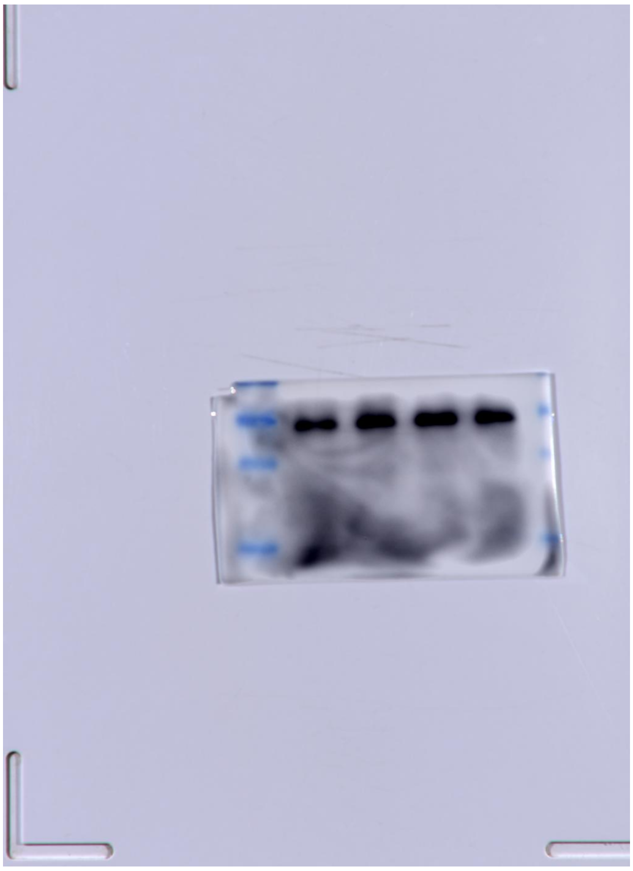
Figure 6C SW1116 cleave-Caspase3

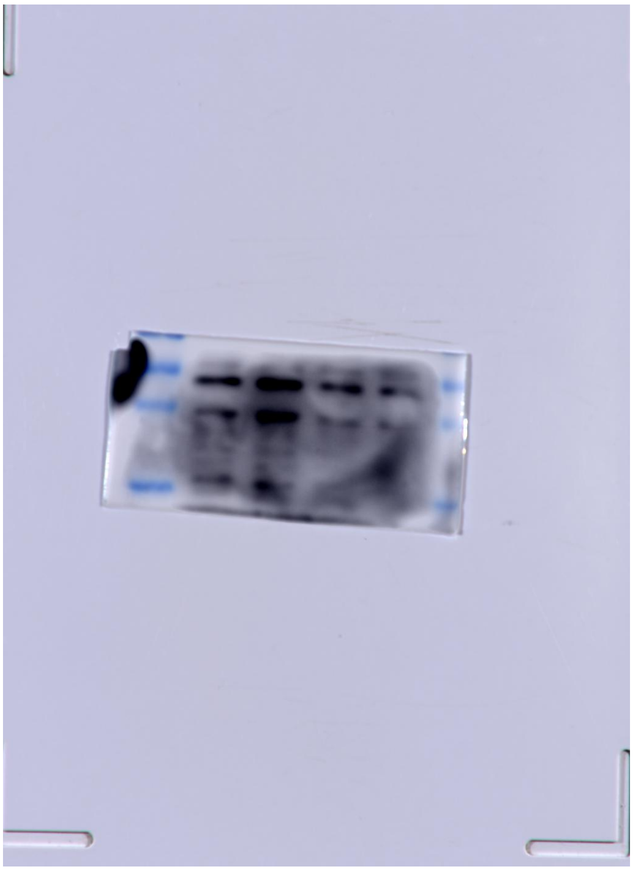
Figure 6C SW1116 β-Tublin

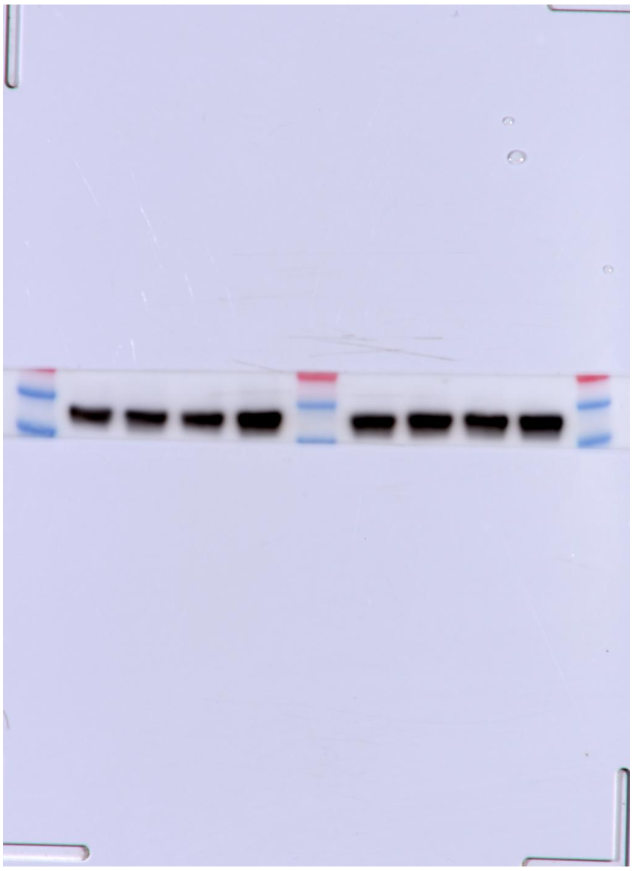

Supplement: Supplementary file 1 — Supplementary Material 1 [file 12885_2023_11077_MOESM1_ESM.pdf]
